# Expression and characterization of an endo-β-1,6-galactanase from *Arabidopsis thaliana*

**DOI:** 10.1042/BCJ20253301

**Published:** 2025-12-17

**Authors:** Koen Gistelinck, Xiaolan Yu, Arthur Leyder, Vinicius J.S. Osterne, Tom Desmet, Theodora Tryfona, Els J.M. Van Damme

**Affiliations:** 1 Laboratory of Biochemistry and Glycobiology, Department of Biotechnology, Ghent University, Ghent, 9000, Belgium; 2 Department of Biochemistry, University of Cambridge, Cambridge, CB2 1QW, U.K.; 3 Centre for Synthetic Biology, Department of Biotechnology, Ghent University, Ghent, 9000, Belgium; 4 Department of Biochemistry and Molecular Biology, Federal University of Ceará, Fortaleza, Ceará, 60440-970, Brazil

**Keywords:** *Arabidopsis thaliana*, endo-β-1,6-galactanase, enzyme specificity, glycosyl hydrolase, molecular dynamics

## Abstract

To date, only fungal and bacterial endo-β-1,6-galactanases from glycoside hydrolase subfamilies GH5_16 and GH30_5 have been characterized. β-1,6-galactan chains are primarily structural components of type II arabinogalactans present in plant and algal cell walls. The *ATIYA1* gene, originating from *Arabidopsis thaliana*, was cloned and expressed in *Komagataella phaffii*. The precursor protein consists of an *N*-terminal signal peptide, a glycosyl hydrolase domain, and a *C*-terminal ricin B-like module. The recombinant enzyme was able to hydrolyze β-1,6-linked galactan chains. Optimal conditions for enzymatic activity were observed at pH 5 and 30°C. The *ATIYA1* gene encodes the first endo-β-1,6-galactanase identified from plants and represents the first characterized member within the GH5_11 subfamily.

## Introduction

Carbohydrates play an important role in different physiological processes of life including growth, development, signaling, biotic, and abiotic stresses [1–8]. Among the structurally diverse carbohydrates in plants, β-1,6-galactan chains mainly occur in type II arabinogalactan proteins (AGPs), representing one specific class of plant glycoconjugates present at the cell wall. AGPs are common to land plants and algae [9–11] and consist of a protein backbone and a complex structure of carbohydrates. After the modification of proline by prolyl 4-hydroxylases, sugar moieties are attached to hydroxyproline. The main component of these glycans is a β-1,3-linked galactan backbone with branches of β-1,6-galactan chains. The structure is modified with several α-l-arabinose residues, hence the name arabinogalactan. Other substitutions, including rhamnose, fucose, xylose, and (4-*O*-methyl-) glucuronic acid, have been reported [7,12–17]. It is noteworthy that only a minor fraction of the plant cell wall is composed of AGPs. Nonetheless, the carbohydrate structures of these glycosylated proteins are associated with several biological processes including growth, reproduction, and (a)biotic stress tolerance [6,7,18]. Despite their importance, plant glycosyl hydrolases that specifically hydrolyze β-1,6-galactan chains in an endo-acting manner have not been identified yet.

Glycosyl hydrolases are key enzymes that hydrolyze glycosidic bonds and have been classified into different families based on amino acid sequence similarity [19,20]. Among them, the Glycoside Hydrolase family 5 (GH5) is one of the largest families with 49.718 recorded members and 33 distinct activities (Carbohydrate-Active enZYmes database in September 2024) [21]. The GH-A clan includes the GH5 family and is characterized by a (β/α)₈ barrel structure, also known as the TIM barrel [22]. GH5 family enzymes contain two conserved glutamate residues that hydrolyze the glycosidic bond via a retaining mechanism. These catalytic residues are present at the *C*-terminal end of β-strand 4 (acid/base) and β-strand 7 (nucleophile). During the retaining mechanism, the nucleophile attacks the anomeric carbon, which gives rise to the glycosyl-enzyme intermediate. In the meantime, the acid/base will act as an acid and protonates the leaving group. In turn, the acid/base activates a water molecule by deprotonation. The deprotonated water molecule attacks the glycosyl-enzyme intermediate, resulting in the release of the carbohydrate that retained its original stereochemistry [21,23–26].

The GH5 family is divided into 55 subfamilies based on sequence similarity [21]. The main activities associated with the GH5 family are endo-β-1,4-glucanase (EC 3.2.1.4), endo-β-1,4-mannanase (EC 3.2.1.78), and endo-β-1,4-xylanases (EC 3.2.1.8). However, several subfamilies have not yet been characterized, such as GH5_11, GH5_20, GH5_24, GH5_30, GH5_32, GH5_33, GH5_42, GH5_49, GH5_50, GH5_51, GH5_54, and GH5_56 [21,27]. Members of the uncharacterized subfamilies have often been misannotated as one of the prevalent activities found in the GH5 family. At present, no information has been reported for GH5_11. This subfamily only occurs in eukaryotic organisms such as plants, fungi, and specific metazoans such as *Adineta vaga* [21]. This lack of biochemical characterization highlights the potential for discovering novel functions within this diverse and widespread enzyme family.

A genome-wide survey of putative lectin sequences in *Arabidopsis thaliana* retrieved two members of the GH5 enzyme family containing ricin B-like lectin domains [28]. Similarly, a comparative analysis of lectin domains across various plant species, including *Glycine max*, *Oryza sativa*, and *Cucumis sativus*, identified several GH5_11 enzymes as multidomain proteins incorporating ricin B-like domains [29]. These findings suggest that the fusion of the GH5_11 and ricin B domains may represent a conserved domain architecture in plants, highlighting plant GH5_11 enzymes as promising targets for further investigation. Moreover, a functional study in rice involving knockout mutants from a GH5_11 enzyme, namely *Low Seed Setting rate 1 (LSSR1*), reported a reduced seed setting rate presumably caused by abnormal pollen germination, pollen tube penetration, and pollen tube growth in the style [30], further adding to the enigmatic nature and importance of this subfamily, and highlighting the need to elucidate their catalytic activity.

*Arabidopsis thaliana* possesses five GH5_11-annotated genes. The aim of this study is to elucidate the activity of the GH5_11 glycosyl hydrolase subfamily through the functional characterization of one of its members, in particular ATIYA1 (encoded by locus AT1G13130) [21]. In this study, we focus on the heterologous expression, purification, and biochemical characterization of ATIYA1. Specifically, the catalytic activity of the enzyme toward various substrates as well as its catalytic efficiency on distinct galacto-oligosaccharides was evaluated.

## Results

### Analysis of GH5_11 sequences

**Figure 1:**
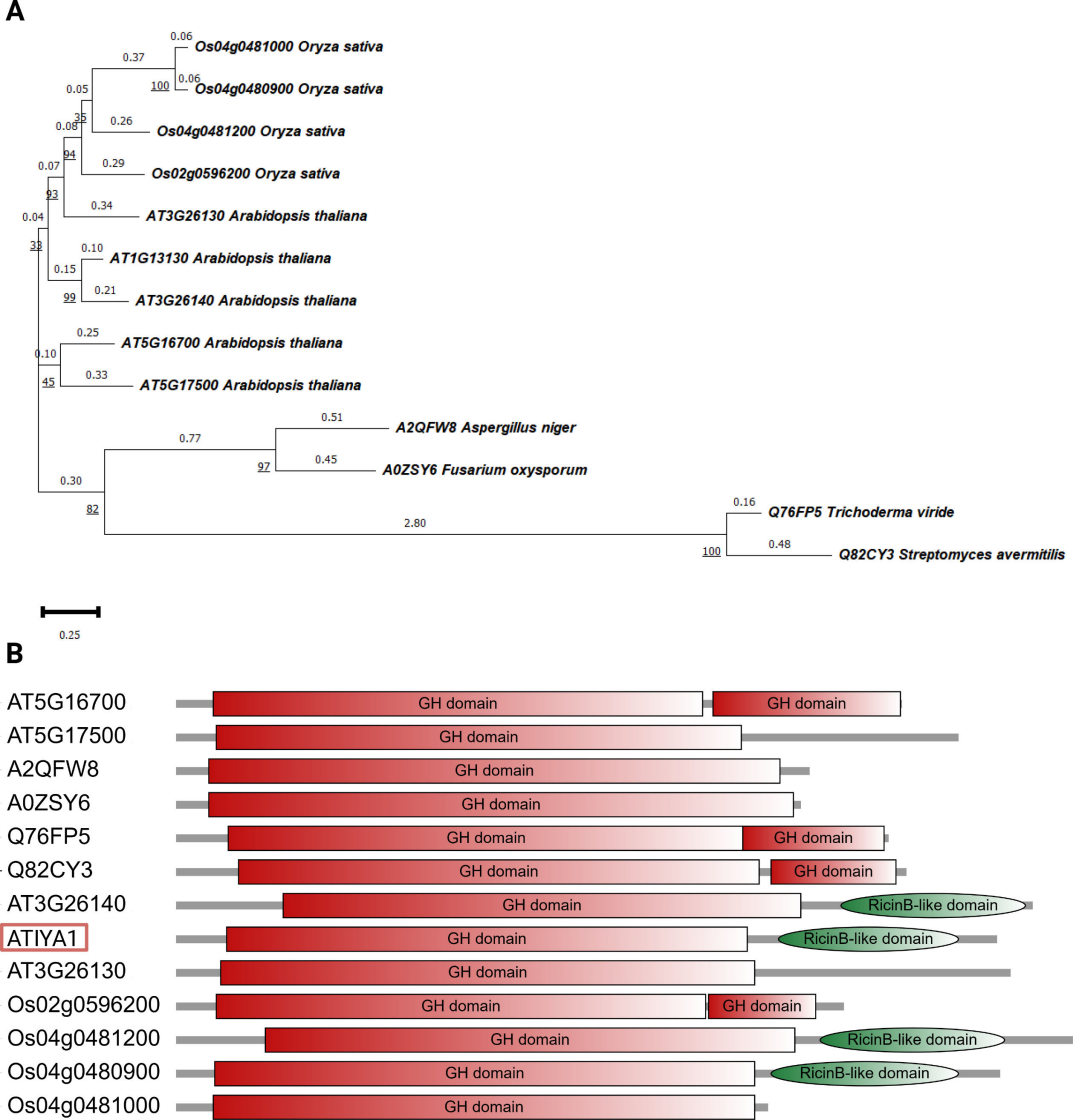
Bioinformatic analysis of GH5_11 proteins from *A. thaliana* and *O. sativa,* and representatives with endo-β-1,6-galactanase activity from GH30_5 and GH5_16. (**A**) Phylogenetic tree of GH5_11 proteins from *A. thaliana* and *O. sativa* and representatives. The phylogenetic analysis is only based on the GH domain containing the TIM-barrel structure. The underlined values describe the bootstrapping values and the values on top of the branches represent the evolutionary distance. (**B**) Domain architecture of GH5_11 proteins and representatives was determined for the complete sequence. The domain architecture was drawn to scale based on amino acid length for each protein, highlighting the relative size and spacing of each protein and its protein domains. The encircled identifier corresponds to ATIYA1, encoded by AT1G13130.

A previous bioinformatics study reported the phylogenetic relationship between the GH5_11 and GH5_16 subfamilies, the latter exhibits endo-β-1,6-galactanase activity, suggesting that the GH5_11 enzymes could exert similar activities [27]. A search in the CAZY database yielded five unique GH5_11 proteins for *A. thaliana* and four sequences for *O. sativa* ssp. japonica. Similar to the reference GH30 endo-β-1,6-galactanase sequences Sa1,6Gal5A (Uniprot ID: Q82CY3) from *Streptomyces avermitilis* and Tv1,6Gal5A (Uniprot ID: Q76FP5) from *Trichoderma viride,* the sequences from *A. thaliana* and *O. sativa* encode multiple domains (Figure 1), such as additional GH domains or ricin B-like lectin domains, next to the core GH domain. The GH30_5 sequences consist of 2 domains, which is a core trait of the GH30 subfamily. Next to the core TIM-barrel structure, the enzymes are also composed of a β-structure [31]. The *Arabidopsis* sequences AT3G26130 and AT5G17500 encode long *C*-terminal peptides, but at present, these domains have not been identified.

Using SignalP v6.0, the ATIYA1 protein is predicted to be synthesized with a signal peptide (likelihood = 0.6714) (Supplementary Figure S1). The 552-amino acid precursor protein, including the signal peptide (SP), has a calculated molecular weight of 61.51 kDa and an isoelectric point (pI) of 8.51.

The PlantPTM viewer 2.0 concludes that at least 1 putative N-glycosylation site with high confidence at position 404 and 3 additional positions with medium confidence at amino acid 69, 75, and 464 occur in the mature ATIYA1 amino acid sequence. Since all asparagine residues in the N-X-S/T sequon are located at the protein surface, the 3D structure of ATIYA1 can carry 4 N-glycans (Supplementary Figure S2).

**Figure 2:**
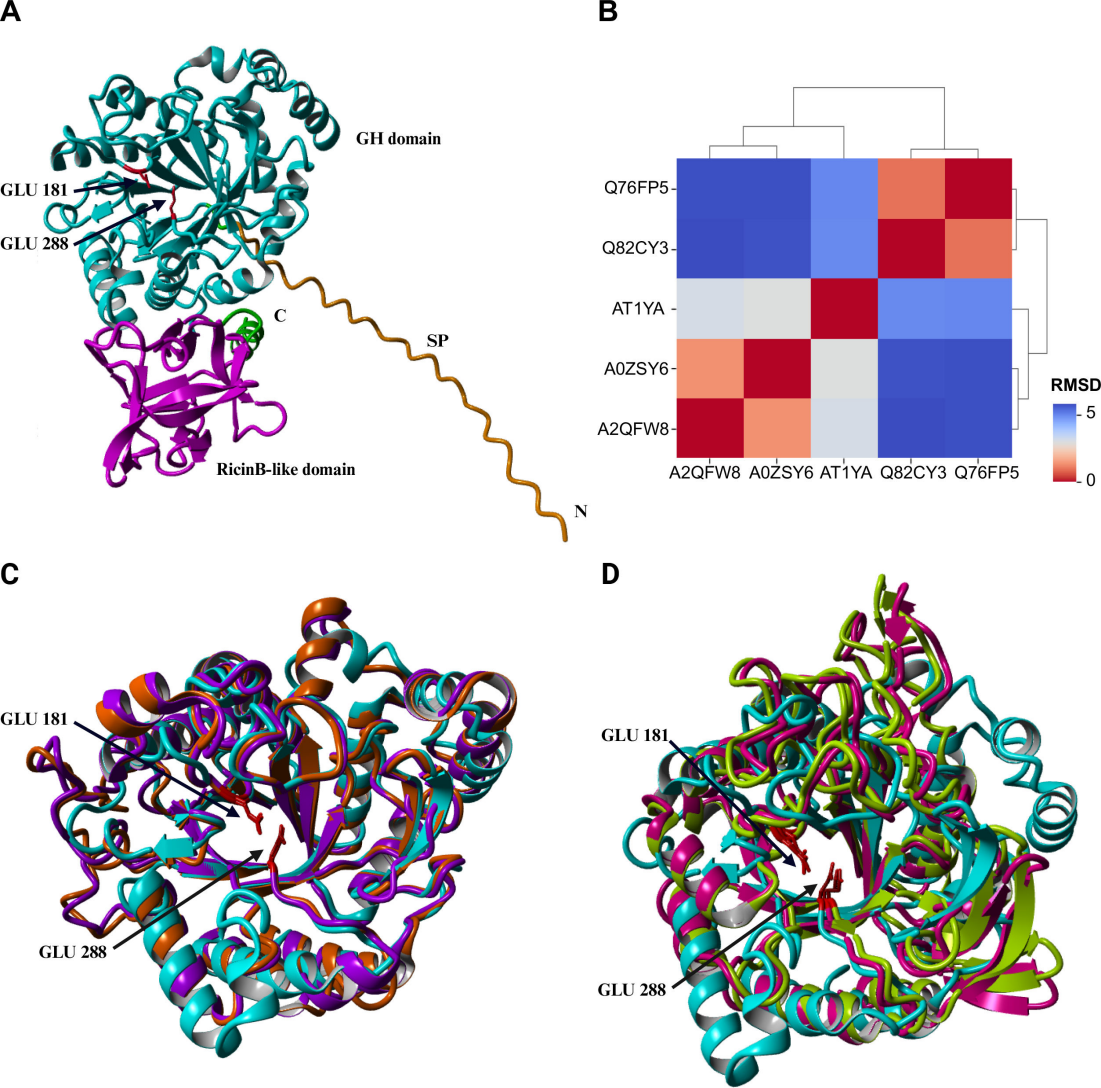
Structural analysis of ATIYA1 and comparison with GH5 and GH30 representatives. (**A**) Overview of the 3D structure of the ATIYA1 precursor protein containing the glycosyl hydrolase (GH domain, cyan), the ricin B-like domain (magenta), the signal peptide (SP, orange). The active glutamic acid residues (red) and the *N*- and *C*-terminus (**N, C**) are indicated. (**B**) Heatmap combined with hierarchical clustering of the structural alignment data from ATIYA1 with the GH5 and GH30 reference proteins. (**C**) Structural alignment of the GH5 representatives A0ZSY6 (purple) and A2QFW8 (orange) with ATIYA1 (cyan). (**D**) Structural alignment of the GH30 reference proteins Q76FP5 (green) and Q82CY3 (pink) with ATIYA1 (cyan). The glutamate residues indicated correspond to the catalytic site residues from ATIYA1

ATIYA1 was compared with various characterized endo-β-1,6-galactanases from both the GH5 and GH30 families. The structural alignment helps to assess structural conservation (Figure 2). Given that both GH5 and GH30 adopt a TIM-barrel architecture, the alignment allows for a direct comparison of their core structural elements, including the positioning of catalytic residues and substrate-binding pockets. The 3D structures of ATIYA1 and the reference proteins are very similar. The root-mean-square deviation (RMSD) values between the characterized endo-β-1,6-galactanases and ATIYA1 were on average 2.99 and 4.95 for the GH5 and GH30 endo-β-1,6-galactanases, respectively.

**Figure 3:**
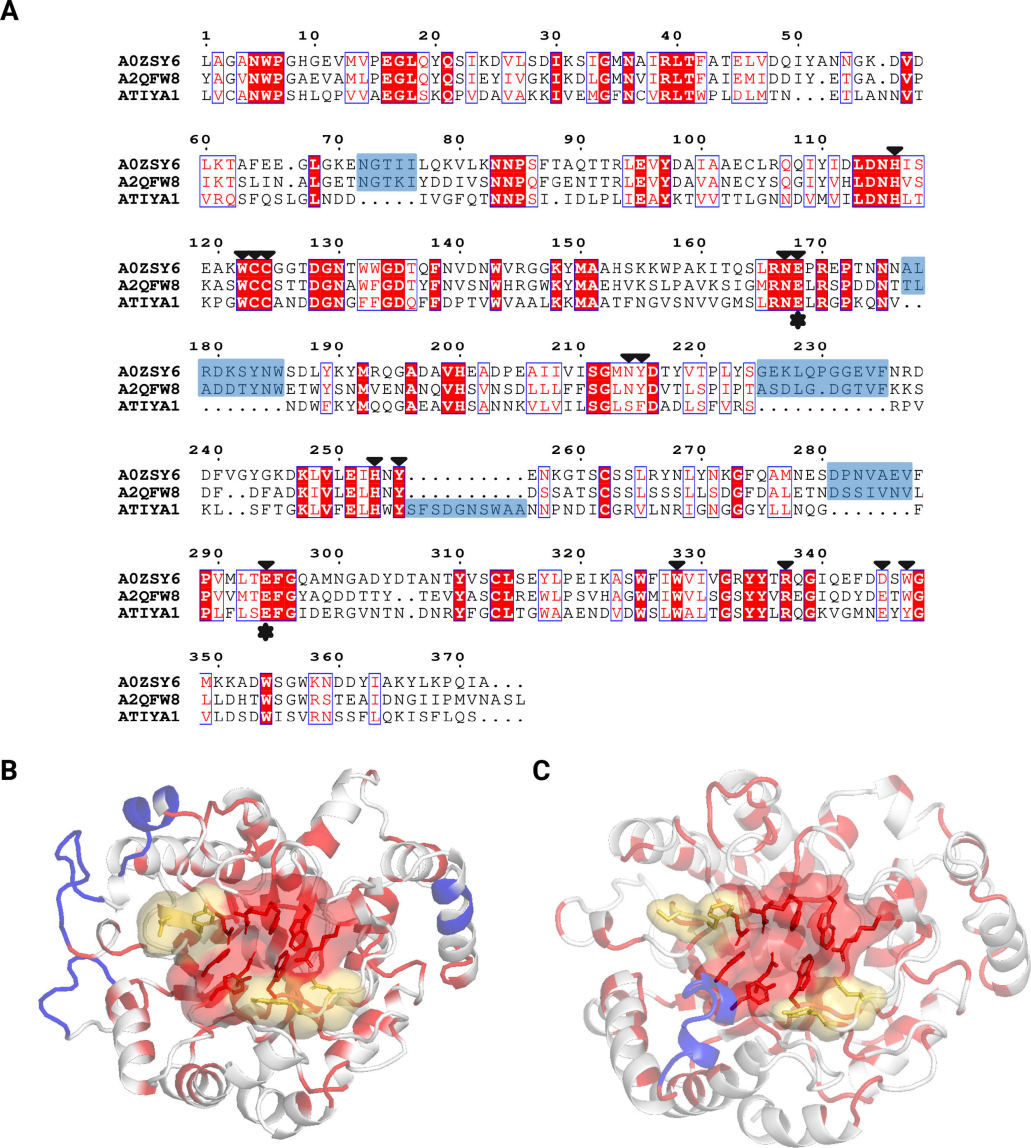
Multiple sequence alignment and structural views highlighting conserved residues for ATIYA1 and GH5_16 representatives. (**A**) Multiple sequence alignment of ATIYA1 with the characterized GH5_16 enzymes An03g01050 (UniProt ID: A2QFW8) and FoGal1 (UniProt ID: A0ZSY6) using MAFFT. Gap regions (insertions ≥ 5 residues) are highlighted in blue. Residues that are identical across sequences are shown in red boxes, whereas similar amino acids (chemically equivalent substitutions) are shown as red letters within boxes. Stars below the alignment indicate the catalytic glutamate residues, and arrowheads above the alignment mark residues involved in substrate binding. Structural representation of FoGal1 (**B**) and ATIYA1 (**C**) showing the spatial distribution of conserved residues. Residues conserved or similar between the enzymes are colored red, non similar residues are shown in white, and gap regions are displayed in blue based on the sequence alignment. Amino acids located within the substrate-binding cleft are represented with sticks, with similar residues within the cleft marked in yellow.

To further evaluate the relationship between ATIYA1 and GH5_16 representatives, An03g01050 (Uniprot ID: A2QFW8) and FoGal1 (Uniprot ID: A0ZSY6), a multiple sequence alignment was generated (Figure 3A). The catalytic glutamate residues are conserved in the GH5 domains. Several residues within the substrate-binding cleft are also identical, while certain peripheral positions display chemically equivalent substitutions (e.g., hydrophobic or polar residues of comparable size and character). The high degree of amino acid conservation surrounding the catalytic center and substrate-binding groove suggests that ATIYA1 could act as an endo-β-1,6-galactanase. Additionally, sequence alignment revealed that GH5_16 enzymes possess four extended loop regions (insertions ≥ 5 residues), but only one such insertion was identified in ATIYA1. To visualize sequence–structure relationships, similar positions were mapped onto the predicted structures GH5_16 enzyme FoGal1 (Figure 3B) and ATIYA1 (Figure 3C).

### Heterologous expression of ATIYA1 in *K. phaffii*

**Figure 4:**
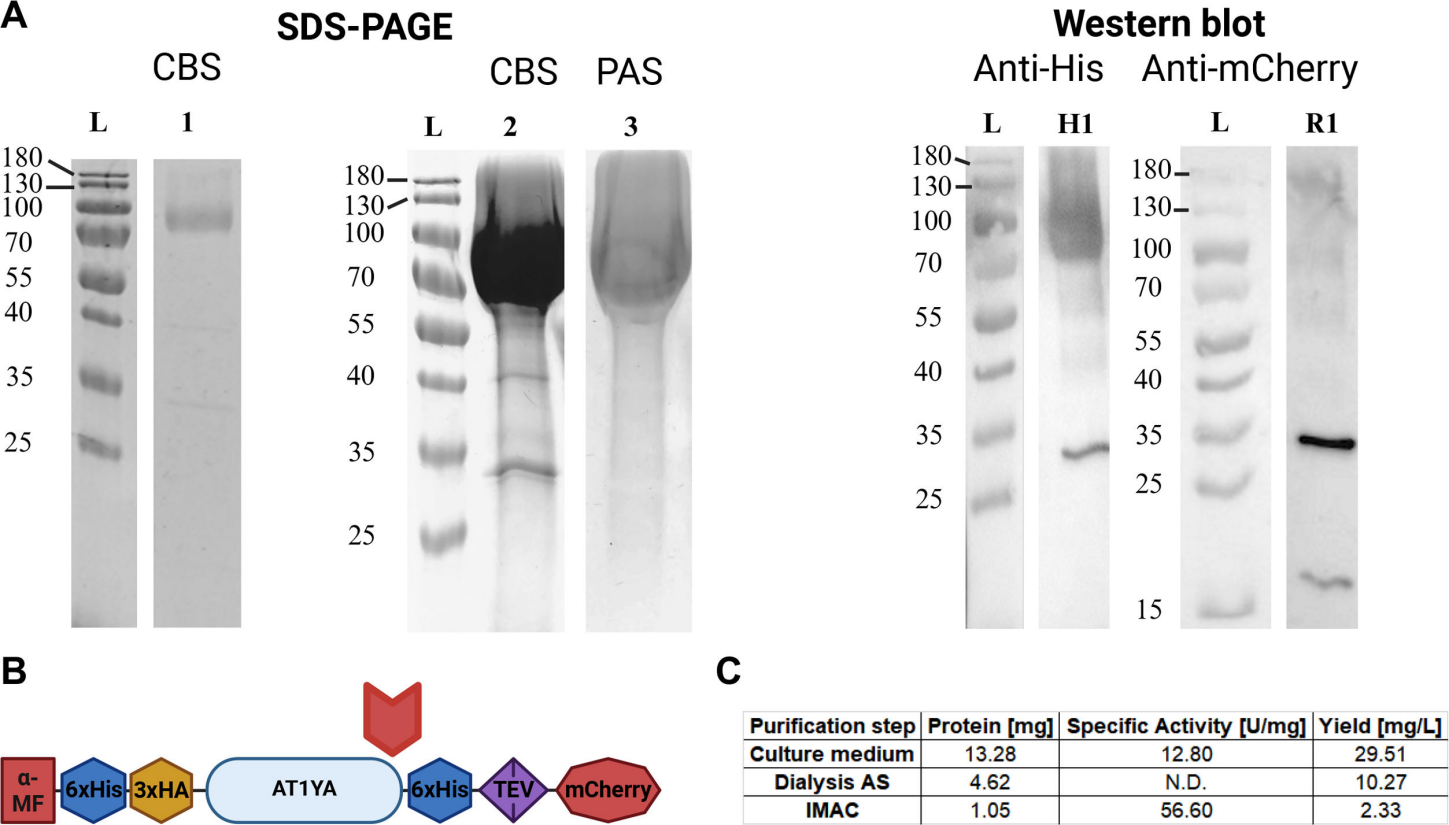
Protein characteristics of recombinant ATIYA1. (**A**) SDS-PAGE and Western blot analysis of ATIYA1 expressed in *K. phaffii*. Different experiments are merged in this image. Lane 1: SDS-PAGE followed by Coomassie staining (CBS) of (~1 µg purified protein); lane 2: SDS-PAGE followed by CBS of 30 µg of ATIYA1; lane 3: SDS-PAGE of 30 µg ATIYA1 after periodic acid-Schiff (PAS) staining to elucidate the presence of glycans. Western blot analysis was performed with different antibodies for 1 µg of purified protein. Lane H1: Western blot analysis using anti-his antibody; lane R1: Western blot analysis using anti-mCherry antibody (Supplementary Figure S10). (**B**) Putative protease cleavage-site indicated by a red arrow. (**C**) Enzyme activity of ATIYA1 during purification. Dialysis AS: The ammonium sulfate-precipitated protein fraction obtained after dialysis. N.D.: not determined.

After SP cleavage, the mature protein consists of 521 amino acids and has a calculated molecular weight of 57.78 kDa and a pI of 8.43. The codon-optimized GH5_11 sequence from *A. thaliana*, ATIYA1 (gene locus AT1G13130), was expressed as part of a fusion protein with the mCherry domain in the methylotrophic yeast *K. phaffii* strain X-33. The recombinant protein consists of 809 amino acids; it encodes a multidomain fusion protein with a molecular weight of 90.48 kDa and a pI of 6.08. The mCherry fluorescent tag facilitated assessing the expression of ATIYA1 in the culture medium. The His6-tagged enzyme was purified using IMAC (Figure 4) and shows a molecular weight of approx. 90 kDa which is in agreement with the predicted size of 90.48 kDa. Western blot analysis using anti-His and anti-mCherry antibodies revealed that the recombinant protein no longer contains the mCherry domain. For both antibodies, an additional band is detected between 25–35 kDa; this band corresponds to the mCherry domain. The cleaved mCherry domain still contains a His6-tag, suggesting that there is a protease cleavage site in the ATIYA1 amino acid sequence. Judging from the size of the recombinant proteins, this cleavage site most likely occurs before the His6-tag *C*-terminally attached to the GH domain (Figure 4B). PAS staining revealed (Figure 4A, lane 2 and 3) that the protein is glycosylated, which confirms the prediction by *in silico* analysis of the sequence. Multiple fractions of the GH5_11 protein obtained during the purification steps were evaluated for enzymatic activity using assays with mildly acid digested gum arabic (DGA) at pH 5 and 30°C (Figure 4C). The specific activity of the protein increased fourfold during protein purification. Overall, these results demonstrate the successful expression and purification of ATIYA1 as a glycosylated and enzymatically active fusion protein.

### Substrate specificity

The activity of the GH5_11 enzyme ATIYA1 was evaluated using a range of polysaccharide substrates (Table 1). The enzyme specifically cleaves β-1,6-galactose linkages and showed highest activity on DGA at pH 5 and 30°C, which was used as a reference. DGA primarily consists of β-(1,6)-, (1,3)- and (1,3,6)-galactosyl linkages with some glucuronic acid and minimal arabinose substitutions [32]. In contrast, substrates such as gum ghatti, gum arabic, larch arabinogalactan, and PSB-D AGP contain β-1,6-galactose chains heavily substituted with arabinose and other monosaccharides, which correlated with lower enzymatic activity. It was hypothesized that these substitutions hinder the access of GH5_11 enzyme to galactan chains. Despite their complex decoration with arabinose and glucuronic acid [32–34], the enzyme still showed some activity on plant-derived AGPs confirming the ability of ATIYA1 to hydrolyze endogenous plant AGPs. Additionally, ATIYA1 displayed limited activity toward noncellulosic glucose polysaccharides (xyloglucan and laminarin) and xylan. However, no activity was detected on mannan-related polysaccharides or carboxymethylcellulose (CMC), which are common substrates for GH5 enzymes. The enzyme also showed no activity on β-1,4-galactan substrates.

**Table 1:** Analyses of substrate specificity of ATIYA1 for several substrates.

Polysaccharides	Main linkages	Main substitutions	Relative activity [%]	Standard deviation [%]
Larch AG	β-1,3 (Gal), β-1,6 (Gal)	α-(Ara)	86.9	4.9
Gum Arabic	β-1,3 (Gal), β-1,6 (Gal)	α-(Ara), β-(GlcA)	1.3	0.5
PSB-D AGP*	β-1,3 (Gal), β-1,6 (Gal)	α-(Ara)	60.1	
β-1,4-galactan	β-1,4 (Gal)	/	-	-
Ghatti gum	β-1,6 (Gal)	α-(Ara)	12.1	2.2
DGA	β-1,6 (Gal)	β-1,3 (Gal), β-(GlcA)	100	
β-1,3-glucan	β-1,3 (Glc)	/	0.8	0.4
CMC	β-1,4 (Glc)	/	-	-
Xyloglucan	β-1,4 (Glc)	α-1,6 (Xyl)	0.7	0.3
Laminarin	β-1,3 (Glc)	β-1,6 (Glc)	4.6	1.0
Galactomannan	β-1,4 (Man)	α-1,6 (Gal)	-	-
Glucomannan	β-1,4 (Man), β-1,4 (Glc)	/	-	-
Arabinoxylan	β-1,4 (Xyl)	α-(Ara)	2.1	0.4
Xylan	β-1,4 (Xyl)	/	8.4	1.3

The enzyme was incubated with the polysaccharides at a concentration of 1.0% (w/v). All enzyme reactions were performed three times in 100 mM sodium acetate buffer pH 5 at 30°C. The activity was expressed as a percentage compared with mildly acid digested gum arabic (DGA). Other substrates were larch arabinogalactan (Larch AG), gum arabic, arabinogalactan proteins extracted from plant cell suspension cultures (PSB-D AGP), ghatti gum, β-1,3-glucan from *Euglena gracilis*, carboxymethylcellulose (CMC), xyloglucan from tamarind, laminarin from *Laminaria digitata*, low-viscosity carob galactomannan, konjac glucomannan, wheat flour arabinoxylan, and beechwood xylan. ‘-’ corresponds to undetected values compared with the reference. ‘*’ only one enzyme reaction was performed due to the low yield for the AGP from the PSB-D cells.

### Optimal pH and temperature properties of ATIYA1

**Figure 5:**
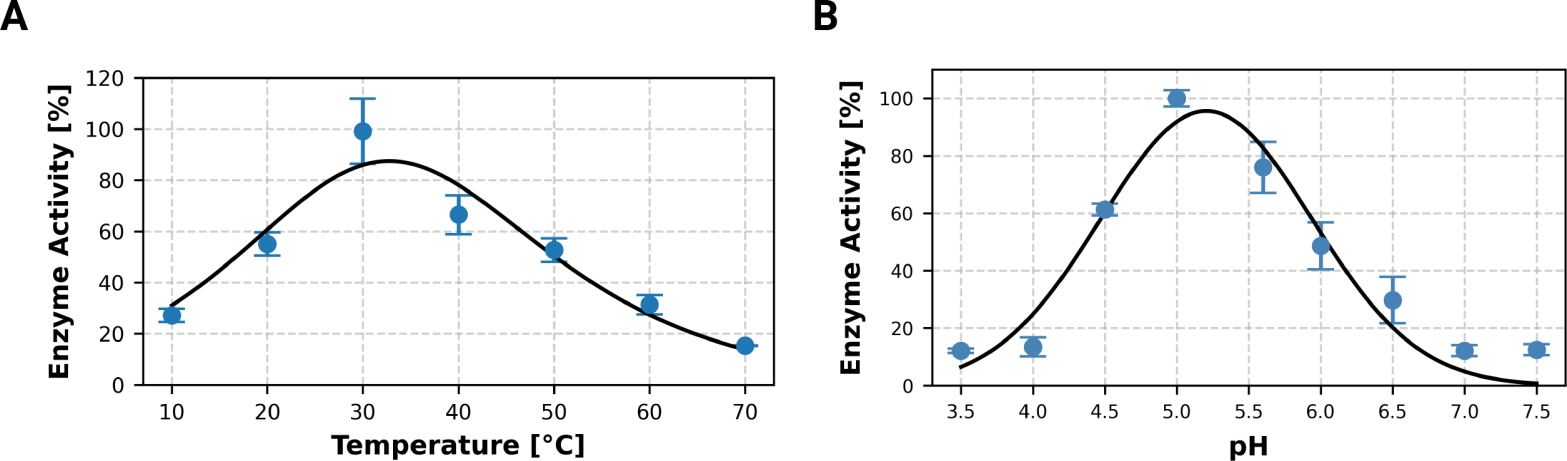
Effect of temperature and pH on the hydrolytic activity of ATIYA1 on DGA. (**A**) Temperature profile of ATIYA1 hydrolysis of DGA. (**B**) pH profile of ATIYA1 hydrolysis of DGA. Enzyme activity was measured in three independent experiments (*n* = 3), and error bars represent the standard error of the mean. For each condition, the activity was normalized to the maximum value observed across the temperature or pH range. A combined Arrhenius and thermal denaturation model was fitted to the temperature data while a Gaussian model was applied to the pH data. Fitted curves are shown as black lines.

The optimal hydrolytic activity of ATIYA1 was determined on DGA. The temperature optimum was assessed across a range of 10 to 60°C, with the highest enzymatic activity observed at approximately 30°C (Figure 5A). In parallel, the pH dependence of the enzyme activity was evaluated over a range of 3.5 to 7.5 and revealed an optimum at pH 5 (Figure 5B).

### Synergistic action of AGP-acting enzymes and ATIYA1

**Figure 6:**
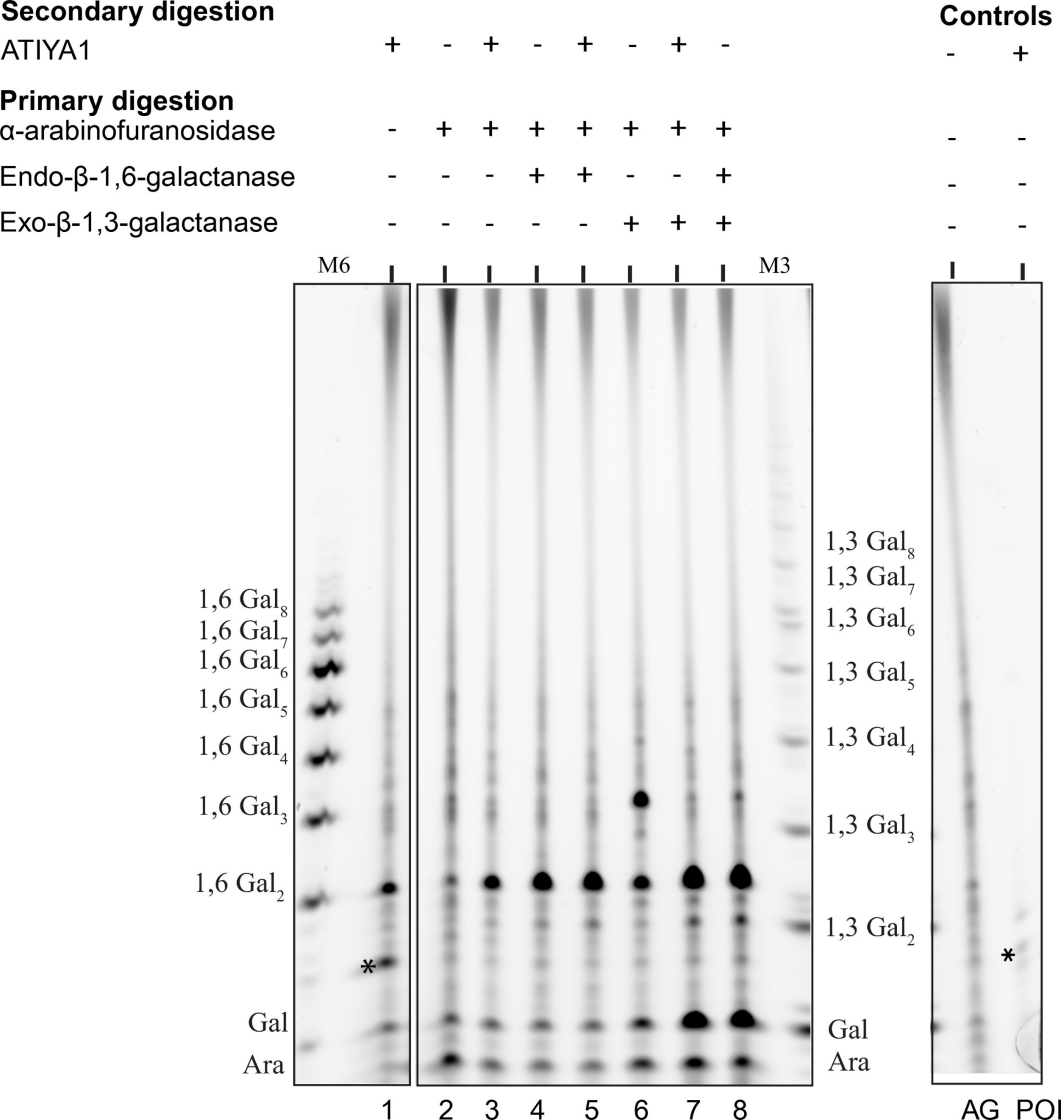
Polysaccharide analysis by carbohydrate gel electrophoresis (PACE) of larch wood AGP hydrolyzed by AG-specific enzymes and ATIYA1. α-l-arabinofuranosidase, endo-β-1,6-galactanase (GH5), and exo-β-1,3-galactanase were used alone or in combination (primary digestions). Primary digestions were terminated by heating at 100°C for 5 min and dried in a centrifugal evaporator. The oligosaccharide products of primary enzymatic treatments were further hydrolyzed with ATIYA1 enzyme (secondary digestion). Minus (-) indicates absence of the enzyme in the cocktail; plus (+) indicates presence of the enzyme in the cocktail. Oligosaccharide ladders prepared from β-(1,6)-galactan (**M6**) and β-(1,3)-galactan (**M3**) were used as migration markers. Larch AG denotes larch arabinogalactan control; POI denotes ATIYA1 enzyme control. Background contaminating band from ATIYA1 enzyme preparation is indicated with an asterisk (*).

Given the structural complexity of AGPs in nature, the synergistic action of different arabinogalactan-degrading enzymes, namely exo-β-(1,3)-galactanase, endo-β-(1,6)-galactanase, and α-l-arabinofuranosidase with ATIYA1 (Figure 6) was evaluated on larch AG.

To evaluate the effect of α-l-Araf substitution on GH5_11 (ATIYA1) digestion, larch AG was first incubated with α-l-arabinofuranosidase, followed by digestion with exo-β-(1,3)-galactanase, endo-β-(1,6)-galactanase, and the ATIYA1 enzyme. The released oligosaccharide fragments were analyzed by PACE.

Hydrolysis of larch AG with ATIYA1 alone resulted in the release of some galactose and an oligosaccharide that co-migrated with the β-1,6-galactobiose standard (Figure 6, lane 1), indicating that ATIYA1 functions as an endo-β-(1,6)-galactanase.

To assess whether α-l-Araf substitutions sterically hinder ATIYA1 activity, we pre-treated larch AG with α-l-arabinofuranosidase before digestion with ATIYA1 (Figure 6, lane 3). This pre-treatment increased the relative intensity of β-1,6-galactobiose, suggesting that the removal of arabinose residues enhances ATIYA1 digestion. The ability of ATIYA1 to hydrolyze α-l-arabinofuranosidase pre-treated larch AGs was comparable with that of the endo-β-(1,6)-galactanase (EC 3.2.1.164) (Figure 6, lane 4). This was further confirmed by additional treatment of α-l-arabinofuranosidase/endo-β-(1,6)-galactanase-pretreated larch AG with ATIYA1 (Figure 6, lane 5), which did not lead to further release of β-1,6-galactobiose.

To further investigate ATIYA1 endo-β-(1,6)-galactanase activity, we pre-treated larch AG with α-l-arabinofuranosidase and exo-β-(1,3)-galactanase (Figure 6, lane 6). The main products of this hydrolysis were β-1,6-galactotriose, β-1,6-galactobiose, arabinose, and galactose. Subsequent hydrolysis with ATIYA1 (Figure 6, lane 7) resulted in β-1,6-galactobiose, arabinose, and galactose, indicating that ATIYA1 cleaved β-1,6-galactotriose into galactose and β-1,6-galactobiose. This demonstrates that ATIYA1 can efficiently hydrolyze β-1,6-galactotriose, a property comparable with that of the endo-β-(1,6)-galactanase (EC 3.2.1.164) (lane 8) when acting on α-l-arabinofuranosidase/exo-β-(1,3)-galactanase-treated larch AG. Taken together, these results indicate that ATIYA1 functions as a β-1,6-galactanase, and the hydrolysis of β-1,6-galactan side chains of AGP is enhanced by the synergistic action with an α-l-arabinofuranosidase.

### Enzymatic degradation of oligosaccharides

**Figure 7:**
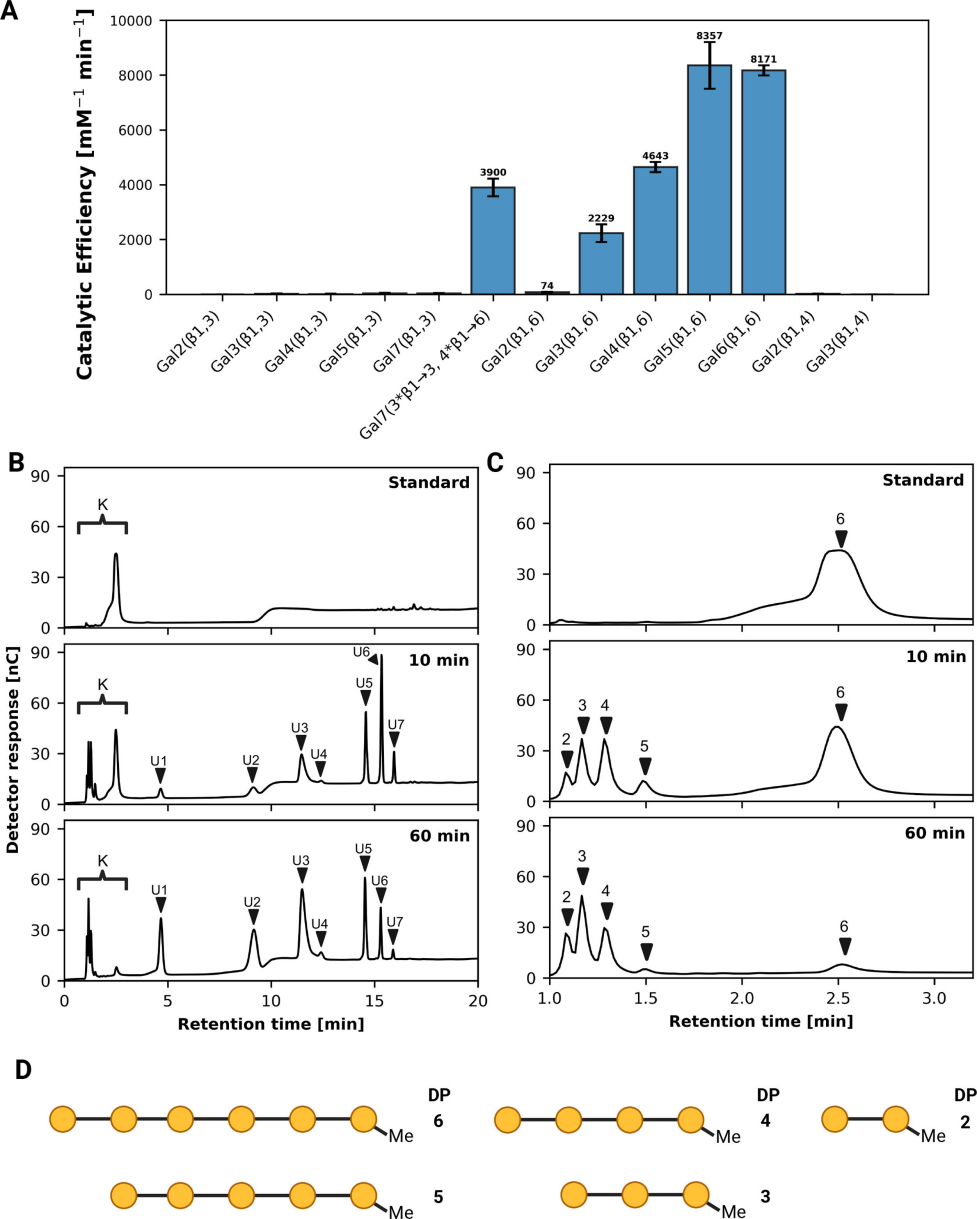
HPAEC-PAD analyses of ATIYA1 activity on methylated galacto-oligosaccharides with varying linkages and degrees of polymerization. (**A**) Catalytic efficiency of ATIYA1 acting on different methylated galacto-oligosaccharides. Linkages are denoted as GalX(β1→Y) or GalX(β1,Y), where X and Y represent the degree of polymerization and the linkage type, respectively. For the β-1,6-linked galacto-oligosaccharides, the catalytic efficiency shown represents the average value. Experiments were performed in triplicate (*n* = 3). Error bars represent the standard error. (**B**) HPAEC-PAD chromatograms of methylated β-1,6-galactohexaoside hydrolysis by ATIYA1: substrate alone (Standard), 10-min, and 60-min reactions (see Supplementary Figure S4 for chromatograms of all time points). Reaction-dependent peaks are labeled as K (known) and U1-7 (unknown). (**C**) Detailed view of region K showing elution of methylated oligosaccharides (1–3 min). Peak identities were assigned based on retention times of reference standards. Arrows 1–6 indicate distinct degrees of polymerization. (**D**) Schematic representation of β-1,6-galactohexaoside and the corresponding hydrolysis products.

The catalytic efficiency of ATIYA1 for multiple methylated β-1,6-; β-1,3- and β-1,4-linked galacto-oligosaccharides with varying degrees of polymerization was determined using Formula 1 (Figure 7A). To illustrate the enzyme’s catalytic activity, the chromatograms of the methylated β-1,6-linked galactohexaoside were selected and are shown for the standard, after 10 and 60 min of incubation (Figure 7B–C). Multiple detector responses were observed following hydrolysis. After incubation, peaks appearing after 4 minutes (Figure 7B **: peaks U1-7**) may correspond to unmethylated oligosaccharides, given the shift of specific peaks over time. However, this remains an assumption as pure unmethylated oligosaccharide standards were not available for confirmation. In contrast, the formation of methylated oligosaccharide fragments (retention time <4 min) ranging from DP2 to DP5 during the early stages of hydrolysis was confirmed using standards of the methylated products (Figure 7C **: peaks 2–6**). The substrate and corresponding hydrolysis products are schematically illustrated in Figure 7D.

The catalytic efficiencies indicate that the enzyme exhibited activity toward the methylated β-1,6-linked galacto-oligosaccharides, preferring higher degrees of polymerization such as methyl-β-1,6-galactopentaoside and methyl-β-1,6-galactohexaoside. The enzyme displayed no detectable activity against β-1,6-galactobiose and no detectable activity against β-1,3/ β-1,4 galacto-oligosaccharides. The enzyme revealed a similar efficiency for the mixed linked galacto-oligosaccharide (Gal7(3*β1→3, 4*β1→6) containing 3 consecutive β-1,3-linked and 4 consecutive β-1,6-linked galactosyl residues compared with the methylated β-1,6-galactotetraose.

### Molecular mechanism and structural analysis via MD simulations

MD simulations were conducted to investigate the dynamic behavior of the free enzyme and the enzyme-ligand complexes. In the initial model prior to the MD simulation, the saccharide chain was arranged to span the active site with an equal number of galactose units extending toward the (−) and (+) subsites. The radius of gyration, a measure of the compactness, for the different systems was calculated (Supplementary Figure S5). Statistical analysis revealed that the ligand-bound states were significantly lower in gyration radius (*P*<0.05), suggesting that the ligand-bound complexes are slightly more compact. In addition, the RMSD values were calculated to see the stability of the complexes (Supplementary Figure S6). Overall, the RMSD values of the different complexes remain clustered within a similar range throughout the MD simulation. Slight increases were observed for both ATIYA1 and the β-1,6-galactohexaose–ATIYA1 complex toward the end of the simulation. However, visual inspection of the trajectory in VMD revealed no obvious structural changes in the protein backbone. Next to the RMSD values, the number of hydrogen bonds (H-bonds) between the ligand and the enzyme was quantified (Supplementary Figure S7). The results reveal a clear distinction among the different ligand-enzyme complexes. The β-1,6-galactohexaose-ATIYA1 complex exhibits on average the highest number of H-bonds, followed closely by β-1,6-galactotetraose displaying a lower count. A substantial reduction in hydrogen bonding was observed in the β-1,6-galactobiose complex.

**Figure 8:**
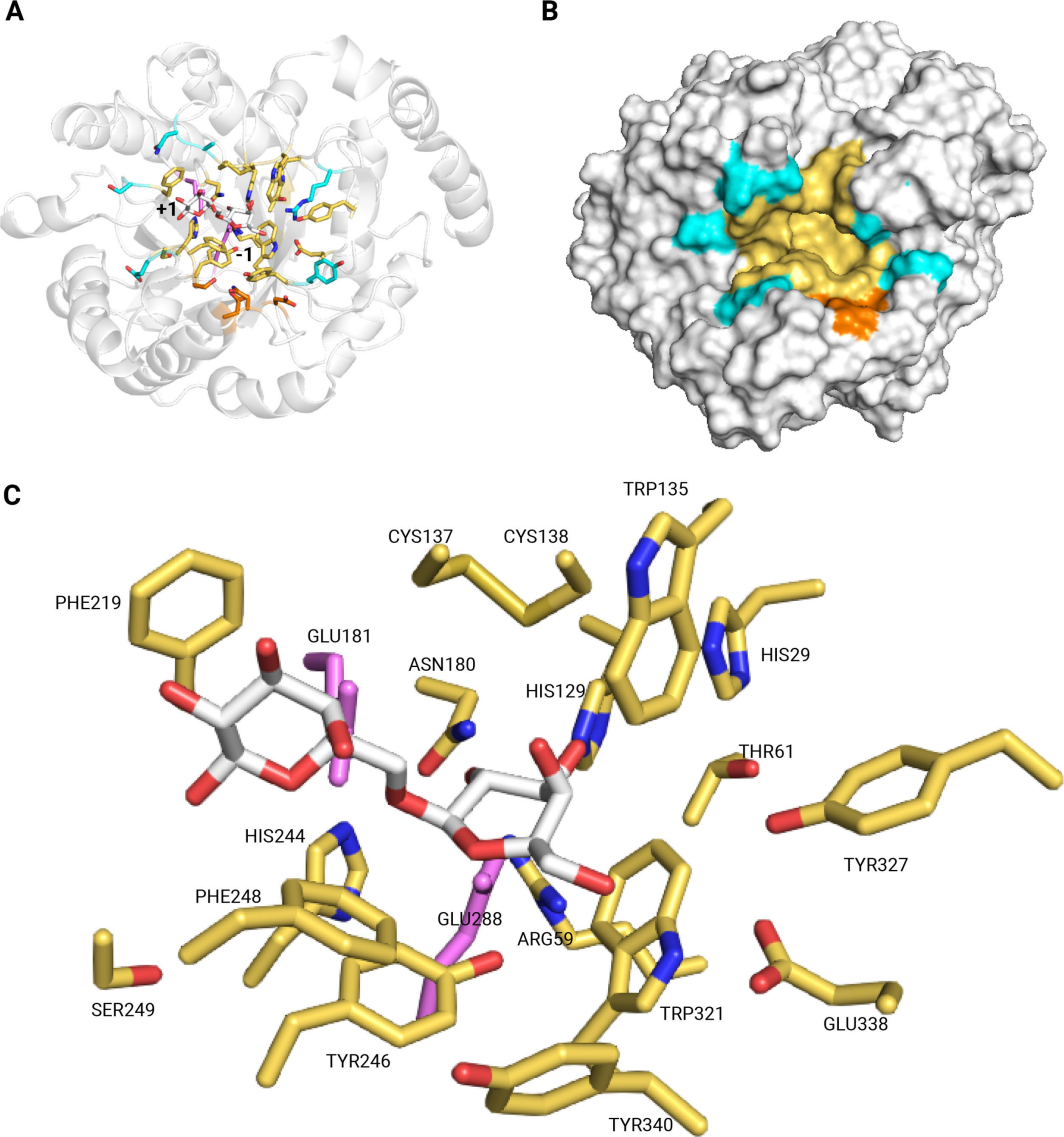
Interacting residues of ATIYA1 with its substrate based on molecular dynamics simulations. Overview of the protein showing the binding pocket (**A**); surface representation (**B**) and detailed view of the β-1,6-galactobiose–enzyme complex (**C**). Residues interacting with β-1,6-galactobiose (yellow) while residues contributing to the extended binding regions for β-1,6-galactotetraose and β-1,6-galactohexaose are depicted in cyan and orange, respectively. The −1 and + 1 subsites are indicated in the overview (**A**). The ligand is shown in white, and the catalytic residues GLU181 and GLU288 are highlighted in violet. Labels in the detailed view identify the interacting residues.

Additionally, residues that persistently interacted with the ligands were identified for the different MD simulations (Supplementary Table S1). The number of interacting residues increased with the degree of polymerization (DP). When the ligand was changed from β-1,6-galactobiose to β-1,6-galactotetraose (Figure 8A), the number of interacting residues increased by approximately 32%. Further increasing the DP from β-1,6-galactotetraose to β-1,6-galactohexaose was accompanied by an additional 20% increase of interacting residues. The interacting residues for β-1,6-galactobiose are shown in detail (Figure 8C). A surface groove that extends from the catalytic site was also observed, providing putative subsites that can accommodate residues of oligosaccharides (Figure 8B).

**Figure 9:**
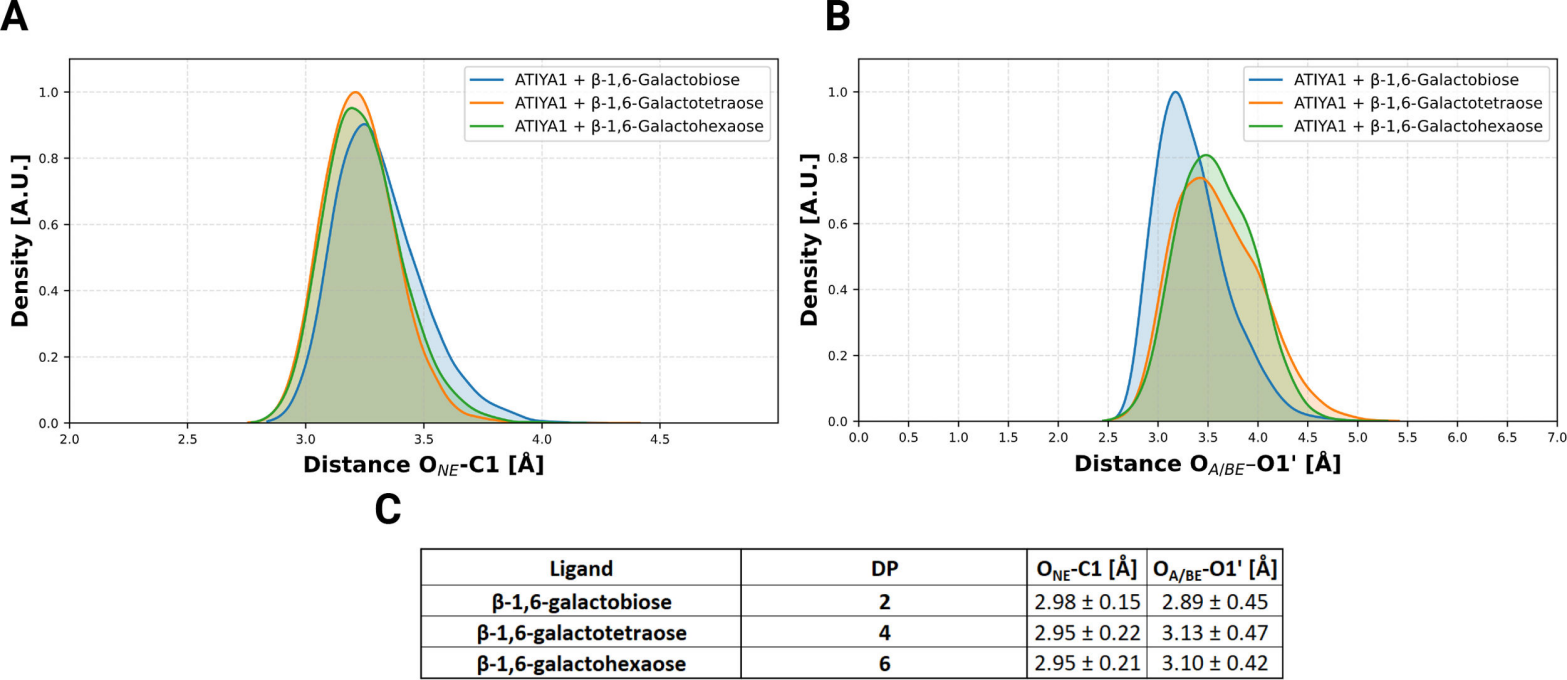
Distance profiles between catalytic residues and key substrate atoms during MD simulations of ATIYA1 and substrates with varying polymerization degrees. Density distribution of the distances between the catalytic residues and the anomeric center (O_NE_-C1) (**A**) and the glycosidic oxygen (O_A/BE_-O1’) (**B**) was calculated throughout the MD simulation for varying degree of polymerization (DP = 2, 4, 6). The average of the distances ( ± standard deviation) is listed in the table (**C**). Density is expressed as arbitrary units (A. U.)

Distances between the catalytic residues were monitored throughout the simulations. The distance between the oxygen from the nucleophile (GLU288) and the anomeric center (O_NE_-C1) and the distance between the acid/base (GLU181) and the glycosidic oxygen (O_A/BE_-O1') were determined throughout the simulations (Figure 9). A single peak was observed for the β-1,6-galacto-oligosaccharide–enzyme complexes for both distances. For the O_NE_-C1 distances, a peak emerged at approximately 3.3–3.6 Å for all systems. In the case of the O_A/BE_-O1′ distances, both the β-1,6-galactotetraose–enzyme and β-1,6-galactohexaose–enzyme complexes exhibited distributions skewed with peaks near 3.5 Å. In contrast, the β-1,6-galactobiose–enzyme complex displays a peak at approximately 3 Å and has a more symmetric distribution.

**Figure 10:**
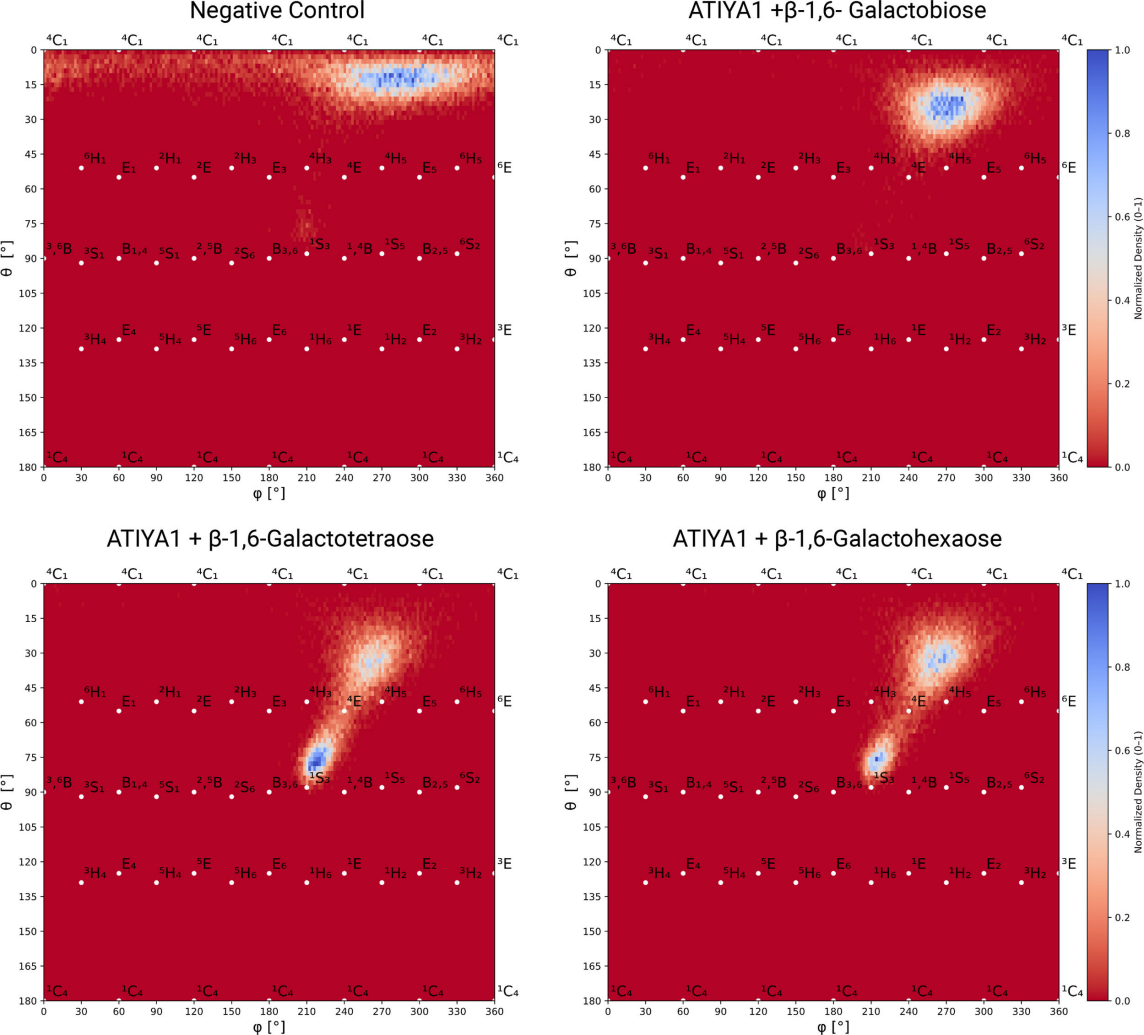
Puckering conformations of the galactose ring at the −1 binding site across The different MD simulations. The puckering of a non-interacting galactose ring was selected as a negative control.

The deformation or the puckering of the galactose ring at the −1 binding site was analyzed across the different MD systems (Figure 10). The Cremer–Pople puckering model was applied to describe the puckering behavior. The negative control galactose ring adopts a ⁴C₁ conformation, corresponding to the canonical chair form typically observed for unstrained sugars. In contrast, increasing the DP promotes a conformational shift in the galactose ring at the −1 binding site, which ultimately favors a ¹S₃ or skew-boat conformation. This puckering behavior suggests that the galactose ring at the −1 binding site undergoes distortion toward a high-energy conformation, consistent with the formation of a transition state during catalysis. Overall, there is a clear shift from the typical ⁴C₁ conformation for all substrate-enzyme complexes toward ^4^E-configuration. However, the skew-boat conformation of the −1 galactose ring is more easily adopted with longer β-1,6-galactan chains, namely DP4 and DP6, suggesting that ATIYA1 may more readily accommodate or stabilize extended substrates during hydrolysis.

To examine the intrinsic ring flexibility of β-1,6-galacto-oligosaccharides in an aqueous solution, molecular dynamics was also performed for free β-1,6-galactohexaose (Supplementary Figure S8). The simulation revealed that the internal flexibility of the oligosaccharide influences the puckering of individual galactose rings: the central residues (positions 3–4, counting from the reducing end) transiently adopted skew-boat conformations, although to a much lesser extent than those observed for the enzyme-bound oligosaccharides (except in the case of galactobiose).

### Binding affinity of the enzyme through affinity gel electrophoresis

**Figure 11:**
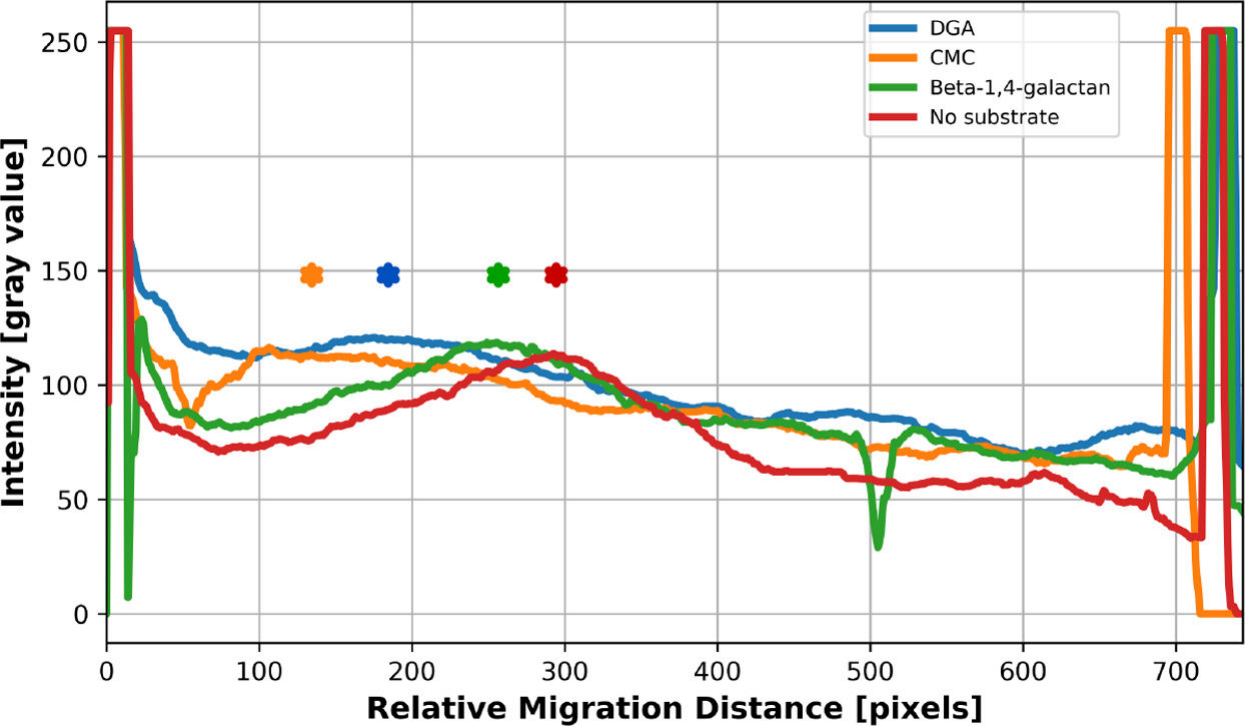
Migration profiles of ATIYA1 in native PAGE gels to evaluate the binding affinity of ATIYA1 for different substrates. The peaks for each profile are indicated with ‘*’.

The binding affinity between ATIYA1 and different substrates was studied through affinity gel electrophoresis. The interaction of ATIYA1 with various substrates, including CMC, DGA and β-1,4-galactan, was initially tested with a substrate concentration of 0.1% (w/v) but no distinct shifts were observed at this substrate concentration (Data not shown). Increasing the substrate concentration of the polysaccharides to 0.3% (w/v) in the native-PAGE gels (Figure 11) showed a significant shift of the enzyme peak with the polysaccharides of CMC and DGA (respectively *P*=0.03605 and *P*=0.0305), suggesting that the protein can interact with these substrates.

## Discussion

This study identifies ATIYA1 from *A. thaliana* as the first plant endo-β-1,6-galactanase and provides biochemical evidence that GH5_11 constitutes a distinct subfamily of β-1,6-galactan active glycosyl hydrolases. These findings suggest that plants have evolved enzymatic mechanisms to remodel type II arabinogalactans, which are polysaccharides central to the architecture and signal transduction in the plant cell wall. The recombinant enzyme was able to specifically hydrolyze β-1,6-galactan chains, displaying maximal activity at pH 5 and 30°C.

We provided evidence that multiple sugar decorations on β-1,6-galactan inhibit the interaction of the enzyme with galactan chains affecting its ability to hydrolyze these compounds. Pre-treatment of larch arabinogalactan with α-l-arabinofuranosidase and exo-β-1,3-galactanase enhanced the degradation of the substrate, indicating that substitutions restrict the access to β-1,6-galactan chains. This is consistent with previous studies showing that α-l-arabinofuranosidase, β-1,6-galactanase, and exo-β-1,3-galactanase act synergistically to enhance the degradation of arabinogalactan [7,16,17]. This synergism confirms that ATIYA1 targets internal linkages within partially de-branched AGPs.

To date, only two proteins with endo-β-1,6-galactanase activity have been characterized in the GH5 family, namely An03g01050 (Uniprot ID: A2QFW8) from *Aspergillus niger* and FoGal1 (Uniprot ID: A0ZSY6) from *Fusarium oxysporum* [35,36]. Several members of the GH5 family have been reclassified to the GH30 family after a detailed sequence and structural study, including some endo-β-1,6-galactanases [31]. Therefore, two GH30 representatives were selected, namely Sa1,6Gal5A (Uniprot ID: Q82CY3) from *Streptomyces avermitilis* and Tv1,6Gal5A (Uniprot ID: Q76FP5) from *Trichoderma viride* [37,38]. However, GH5_16 and the GH5_11 sequences are more related to one another. The sequence similarity between GH5_11 and GH5_16 was reported before [27]. Sequence alignment of the GH5_16 representatives and ATIYA1 further revealed a conserved cluster of amino acids in the catalytic site, including the GLU residues essential for hydrolysis. In addition, comparison of the interacting residues identified in the MD simulations with conserved or equivalent positions among GH5 endo-β-1,6-galactanases revealed substantial overlap (Supplementary Table S2). Approximately 78% of the residues interacting with β-1,6-galactobiose coincide with conserved positions, suggesting that these enzymes share a largely similar substrate-binding architecture.

Domain analysis revealed that several GH5_11 sequences from *O. sativa* and *A. thaliana* encode multi-domain proteins. In addition to the GH domain, the ricin B-like domain or long peptides with unknown function are present, as shown in previous genome-wide studies focusing on lectin domains [28,29]. ATIYA1 consists of the GH5_11 domain and the ricin B-like domain. In this study, we successfully expressed the full protein. However, to date, we have been unable to express the individual domains.

The calculated molecular weight of the protein was consistent with the observed molecular weight determined through SDS-PAGE. However, we detected that the expressed ATIYA1 enzyme no longer contains the mCherry-tag, suggesting the presence of a protein cleavage site that releases the 25–35 kDa mCherry-tag. PAS staining confirmed glycosylation of the recombinant protein. The expression of N-glycosylated proteins in *K. phaffii* typically leads to hypermannosylated N-glycans often resulting in up to 40 mannose residues in the N-glycan structure [39]. These findings suggest that protein cleavage site and N-glycosylation play significant roles in the post-translational modifications of the protein, which may influence its structure and function.

Affinity gel electrophoresis suggested that the recombinant enzyme of interest can interact with both CMC and DGA. It is peculiar that the enzyme is able to interact with CMC, a substrate with a backbone consisting mainly of β-1,4-linked glucose with carboxymethyl groups, since the enzyme did not display any activity toward this substrate. Whether or not the binding of CMC is the result of the interaction of the binding site of the enzyme, the putative binding of the ricin B-like domain, or the result of a newly formed binding site arising from domain fusion remains to be determined.

The data for enzymatic activity were compared with the activities of previously reported endo-β-1,6-galactanases (Supplementary Table S2). ATIYA1 displays similar optima as the GH30_5 endo-β-1,6-galactanases from *S. avermitilis*. The pH optimum for all identified endo-β-1,6-galactanases is at acidic pHs ranging from 3 to 5.5. The pH in plant cells varies for each cell compartment. Whereas the extracellular space has a pH range of 4–5.7, the pH in the intracellular space including the endomembrane system ranges around pH 6–7.5 [40,41]. Taking into account that the protein is synthesized with a putative signal peptide, it is likely to follow the secretory route and can be secreted into the apoplast. The recombinant ATIYA1 shows enzymatic activity at the pH range corresponding with the pH in the apoplast. In contrast to the pH optima, temperature optima for the endo-β-1,6-galactanase activities are diverse and range between 30 and 60°C. The temperature optimum of the recombinant ATIYA1 is slightly higher than the standard growth temperature range of *A. thaliana* which is 16–25°C [42].

Although the use of DGA to determine β-1,3-galactanase and/or β-1,6-galactanase activity is not common, it has been employed in the characterization of various enzymes capable of hydrolyzing β-1,6-galactosyl or β-1,3-galactosyl linkages [32,43–45]. The multiperspective approach used to elucidate the activity of ATIYA1, combining PACE analysis, HPAEC-PAD, and colorimetric assays for reducing sugars, revealed endo-β-1,6-galactanase activity. The substrate analysis also demonstrated that ATIYA1 has some promiscuous activity toward laminarin, arabinoxylan, and xylan. This substrate promiscuity has also been reported for other endo-β-1,6-galactanases. For instance, the GH30_5 enzyme from *Penicillium subrubescens* was reported to be slightly active on wheat bran (xylan), but this was attributed to the potential presence of type II arabinogalactan in the substrate [46]. It is also plausible that the GH5_11 enzyme requires the presence of arabinose residues to act as an arabinoxylanase similar to members of the GH5_34 subfamily [47]. Additional evidence is needed to confirm the aforementioned hypotheses. POGAL30, a GH30_5 from *Penicillium oxalicum*, was able to degrade both laminarin and β-1,3-glucan [45]. Unfortunately, no extra information is provided concerning the promiscuous activity of the known GH5-related endo-β-1,6-galactanases.

To elucidate whether ATIYA1 functions as an endo-acting enzyme, both structural features and product profiles were examined. Structural analysis of the solvated ATIYA1 model shows that the binding site displays a binding groove/cleft which could accommodate multiple galactosyl residues. A binding groove is often associated with endo-acting mechanism [24,48]. In addition, the most crucial piece of evidence supporting that ATIYA1 is an endo-acting enzyme is the simultaneous appearance of multiple lower range DP oligosaccharide peaks after early stage hydrolysis (Supplementary Figure S4). The product distribution is characteristic of endo-acting enzymes and corresponds to the DPr-type product profile described in the refined nomenclature of Leaerts et al. [49].

Structural bioinformatics approaches, including molecular docking and molecular dynamics, were used to study the molecular and mechanistic properties of ATIYA1. Since no structural data are currently available for ATIYA1 or its closest GH5_16 homologs, the initial protein model was obtained from the AlphaFold Protein Structure Database. After solvation of the protein structure, 50 independent molecular docking runs were performed for each ligand, and only complexes displaying a –1/ +1 subsite arrangement characteristic of retaining glycosyl hydrolases were kept for further analysis. Substrate placement at the catalytic –1 and +1 subsites can therefore be considered reliable. Moreover, the galactose residues at the distal subsites for DP4 and in part for DP6 generally remained aligned along the binding groove and did not diverge from the binding site (Supplementary Figure S9). However, in the absence of experimental structural data, caution remains warranted when interpreting the generated data from the MD simulations.

MD simulations provided structural and dynamic insights that may help rationalize the biochemical behavior of the enzyme. Simulations were conducted on β-1,6-galactosyl compounds with increasing DP. Key parameters assessed during the simulations, including the number of interacting residues, number of hydrogen bonds, and ring puckering conformations, highlight the enzyme’s preference for substrates with higher DP. The increase in the number of interacting residues and number of hydrogen bonds with longer oligosaccharides indicates enhanced ligand stabilization via additional binding interactions at putative extended binding sites. Puckering analysis partially supports the preference of the enzyme for higher DP ligands, showing that increased DP (4 and 6) facilitates more effective distortion of the −1 galactose ring toward a transition-state-like conformation. Notably, the ring adopts a ¹S₃ (skew-boat) conformation, a feature also observed in other GH5 enzymes [50,51] and several β-galactosidases from the GH-A clan [52], suggesting that the ligand in the newly characterized enzyme likely assumes the same ¹S₃ conformation during hydrolysis. However, the interatomic distances observed in the MD simulation generally fall within the experimentally suggested ranges for catalytically competent configurations, approximately 2.6–3.5 Å between the nucleophile and the anomeric carbon, and 2.5–3.0 Å between the acid/base and the glycosidic oxygen [53–56]. For DP4 and DP6 systems, the distances between the acid/base and the glycosidic oxygen exceeded these ranges, which could indicate less favorable positioning for hydrolysis. Nevertheless, kinetic analyses revealed a general trend of increasing catalytic efficiency with higher degrees of polymerization, indicating that ATIYA1 preferentially acts on longer β-1,6-galacto-oligosaccharides.

Our findings will assist future studies on type II arabinogalactan proteins and their significance in plant development and tolerance. Currently, several hypotheses have been suggested concerning the importance of plant AGPs for plant fertility. AMOR, a disaccharide found at the nonreducing termini of β-1,6-galactan in AGP carbohydrate chains [17,57], is implicated in the competency of the pollen tube to respond to ovular guidance such as the LURE peptides in *Torenia fournieri* [57]. The ability of ATIYA1 to release β-1,6-galacto-oligosaccharides suggests a putative role of the enzyme to generate AMOR-like glycans. In line with this, the aberrant pollen tube growth in the LSSR1 knockout mutants [30] can be explained by the absence of these AMOR saccharides due to the lack of endo-β-1,6-galactanase activity. Overexpression of a *T. viride* endo-β-1,6-galactanase in *A. thaliana* reduced the length of β-1,6-galactan side chains, thereby affecting cellulose synthesis and deposition. Induced expression of the fungal galactanase in *A. thaliana* influenced seedling development, altering the mechanical properties of the hypocotyl and inhibiting hypocotyl growth in darkness [58]. Whether ATIYA1 will elicit similar *in vivo* effects remains to be investigated.

Overall, the discovery of ATIYA1 opens new avenues for exploring the role of endogenous endo-β-1,6-galactanases in modulating AGP structure, cell wall architecture, and signaling. Together, our findings define GH5_11 as a new glycoside hydrolase subfamily with endo-β-1,6-galactanase activity.

## Materials and methods

### Materials

Most chemicals were of analytical grade and were obtained from Merck (Darmstadt, Germany), AnalytiChem (Zedelgem, Belgium) or MP biomedicals (Eschwege, Germany). β-1,4-galactan (BioSynth, Berlin, Germany), d-cello-oligosaccharides (Elicityl, Crolles, France), arabinoxylan from wheat flour and xylan from beechwood (Neogen, Bray, Ireland) were purchased from different companies.

All methyl-β-glycosyl galacto-oligosaccharides were kindly provided by Prof. Em. Pavol Kováč (National Institutes of Health/National Institute of Diabetes and Digestive and Kidney Diseases (U.S.A.)) [59–62].

Mildly acid digested gum arabic (DGA) was generated as described by Zhang et al. [45] with minor modifications. Gum arabic (18 g) was dissolved in 90 mL of distilled water. The mixture was heated and continuously stirred in a water bath at 95°C. Once the gum arabic was completely dissolved, 10 mL of 2.5 M TFA was mixed with the gum arabic solution. The solution was kept at 95°C for 2 h while stirring. Afterward, the mixture was cooled for 5 min, and the pH was adjusted to pH 7.0 using NaOH. The digested gum arabic solution was centrifuged at 4500 rpm for 10 min at room temperature. The supernatant was dialyzed against distilled water for approximately 16 h (molecular weight cutoff 6–7 kDa). The saccharides were precipitated overnight by 1.5 volumes of anhydrous ethanol. After centrifugation for 20 min at 4500 rpm and 4°C the supernatant was removed, and the pellet was dried.

Arabinogalactan proteins (AGP) were extracted from two-week-old *Arabidopsis* cell suspension cultures (PSB-D). Therefore, 30 mL of one-week-old PSB-D cells were transferred in 270 mL of MSMO medium (4.43 g/L MSMO (minimal organics Sigma #M6899), 30 g/L sucrose, 0.5 mg/L NAA, and 0.05 mg/L kinetin adjusted to pH 5.7 with KOH). The cell culture was grown at 25°C, shaking at 130 rpm in darkness. 1 L of culture was used for the extraction of *Arabidopsis* AGPs, using a protocol adapted from Tryfona et al*.* [17].

### *In silico* analysis of ATIYA1

The isoelectric point (pI) and the molecular weight of the protein were empirically determined (https://web.expasy.org/compute_pi/) [63]. The presence of the signal peptide was evaluated using SignalP version 6.0 [64] (parameters ‘Eukarya’ and ‘Slow output’). Putative glycosylation sites were evaluated using the PlantPTM viewer 2.0 [65]. In addition, the glycosylation sites were evaluated using the 3D model of ATIYA1. Charmm-GUI was utilized to perform *in silico* glycosylation [66–69].

GH5_11 sequences for *Oryza sativa* subsp. japonica and *Arabidopsis thaliana* were extracted from the CAZY database [21]. Characterized endo-β-1,6-galactanases from the GH5 and GH30 families were selected for sequence analysis. CANDy was employed for an automated analysis of domain architectures of the sequences under study and for creating a phylogenetic tree using MAFFT [70]. The phylogenetic tree was constructed only for sequences encoding the GH domains. The truncated sequences were generated by removing additional domains/peptides and signal peptides manually. The phylogenetic tree was visualized using MEGA11 [71]. In addition, GH5 domain amino acid sequences of the characterized GH5_16 enzymes and ATIYA1 were aligned using MAFFT v.7 with default settings [72,73]. The resulting alignment was visualized with ESPript [74,75].

The three-dimensional structures of ATIYA1 and the reference proteins from family GH5 and GH30 were obtained from the AlphaFold Protein Structure Database [76,77]. Model quality measures were evaluated (Supplementary Figure 10). Based on the sequence analysis, the GH domains were extracted and used for structural alignment using the ‘cealign’ algorithm in Pymol v.2.5.2 [78].

### Gene cloning and transformation

The sequence encoding ATIYA1 was codon-optimized for *Komagataella phaffii* and synthesized by GeneArt (Thermo Fisher Scientific, Waltham, MA, U.S.A.). The sequence was amplified and extended with the proper cloning sites through polymerase chain reaction (PCR) using Q5® High-Fidelity DNA Polymerase (NEB, Ipswich, Massachusetts, U.S.A.) and primers A207 (forward primer: AGAAGTGAAGCTTGGTCTCAGGCTCCAGCTACCCTTTGTCCACTTCCTC) and A208 (reverse primer: AGGGCGAGAATTCGGTCTCACTGACAATGGTCTGGTGGCCTTGATGATC). The cycling parameters were 98°C—60 s; 30× (98°C—10 s; 70°C—30 s; 72°C—45 s); and 72°C—2 min. The construct was subcloned into the Golden Gate entry vector pGGC000 using Gibson assembly. Later, the coding sequence was subcloned into a Golden Gate compatible pPICZα-A vector (Supplementary Figure 11).

The pPICZα-A vector was transformed into Top10 *Escherichia coli* cells through heat shock, and transformed cells were selected on antibiotic selective medium containing 25 µg/mL Zeocin and analyzed by colony PCR and DNA sequencing. The plasmid vector was purified using the GeneJET Plasmid Miniprep Kit (Thermo Fisher Scientific, Waltham, MA, U.S.A.) and subsequently linearized using PmeI (NEB, Ipswich, MA, U.S.A.). The linearized plasmid was used to transform competent *K. phaffii* strain X-33 cells through electroporation. The protein production was screened through small-scale expression and subsequent measurement of fluorescence originating from the mCherry tag using a TECAN plate reader (Infinite 200, Tecan, Mannedorf, Switzerland). The best-producing colony was selected for further analysis.

### Protein expression and purification

Cells were inoculated in a 5 mL yeast peptone dextrose (YPD) medium (10 g/L yeast extract, 20 g/L peptone and 20 g/L d-glucose) containing 250 µg/mL Zeocin (Invivogen, San Diego, CA, U.S.A.) and incubated at 28°C at 200 rpm with continuous shaking. The overnight culture was inoculated (1%) in 450 mL buffered glycerol complex (BMGY) medium (1% (w/v) yeast extract, 2% (w/v) peptone, 1.34% (w/v) yeast nitrogen base without amino acids, 0.1 M potassium phosphate buffer pH 6.0, 1% (v/v) glycerol) containing 250 µg/mL Zeocin in six separate 500 mL baffled flasks. The cultures were incubated at 28°C with continuous shaking at 230 rpm for 48 h. Afterward, the cultures were centrifuged at 3871 ***g*** for 6 min. The supernatant was discarded and the pellet of each flask was resuspended in 75 mL of buffered methanol complex (BMMY) medium (1% (w/v) yeast extract, 2% (w/v) peptone, 1.34% (w/v) yeast nitrogen base without amino acids, 0.1 M potassium phosphate buffer pH 6.0, 1% (v/v) methanol) and incubated for 48 h at 28°C and 230 rpm. Every 8–12 h, 1% (v/v of the culture medium) methanol was added to the culture medium. After 48 h incubation, the culture was harvested. Cells were centrifuged for 6 min at 3871 ***g***. The cells were discarded, and the supernatant was used for ammonium sulfate precipitation (80% saturation) of proteins by overnight incubation at 4°C. The proteins were precipitated by centrifugation for 20 min at 8000 ***g*** and 4°C. The supernatant was discarded and the pellet was resuspended in 10 mL of 1 M Tris.HCl (pH 8.0), followed by dialysis against 50 mM Tris.HCl (pH 8.0) to a Brix% of ≤ 5%.

The His6-tagged protein was purified by a nickel-nitrilotriacetic acid (Ni-NTA) affinity chromatography or Immobilized Metal Affinity Chromatography (IMAC). The Ni-NTA Agarose (2 mL, MCLAB, South San Francisco, CA, U.S.A.) was washed three times with 10 mL water and three times with 10 mL equilibration buffer (50 mM Tris.HCl, pH 8.0) before the protein solution was loaded on the matrix. The protein solution was incubated for at least 1 hour while there was a flow of at most 1 mL/min. The matrix was first washed with 50 mM Tris.HCl (pH 8.0) till an OD at 280 nm of less than 0.1 was reached. Then the second wash was performed with 50 mM Tris.HCl (pH 8.0) containing 15 mM imidazole till an OD at 280 nm of less than 0.1 was reached. The proteins were eluted with the elution buffer (50 mM Tris.HCl (pH 8.0) containing 250 mM imidazole). The buffer was exchanged to 50 mM 3-(N-morpholino)propanesulfonic acid (MOPS) (pH 7.0) through dialysis.

The protein content was determined using the Bradford assay (Protein Assay Dye Reagent Concentrate, Bio-Rad, CA, U.S.A.). Bovine serum albumin was used to create a standard curve. Sodium dodecyl sulfate–polyacrylamide gel electrophoresis (SDS-PAGE) using 15% polyacrylamide gels, followed by either Coomassie staining or Western blot analysis, was performed to assess the purity and molecular weight of the proteins. The PageRuler™ Prestained Protein Ladder (Thermo Fisher Scientific) was used as a molecular weight marker.

Western blot analysis was performed as described by Chen et al. [79]. For mCherry detection, the original protocol was followed without modification. The mCherry tag was detected using an anti-RFP monoclonal antibody (ChromoTek, 6G6; 0.4  µg/mL). For the His6-tag detection, the protocol was slightly modified to accommodate buffer composition adjustments. The blocking buffer consisted of 50  g/L non-fat dry milk in PBS (8.5 g/L NaCl, 1.4 g/L Na₂HPO₄, 0.2 g/L NaH₂PO₄; pH 7.4). The washing buffer contained 0.05% (v/v) Tween-20 in PBS. Antibodies were diluted in antibody dilution buffer, consisting of 1% (w/v) BSA in washing buffer. The primary antibody used for His6-tag detection was THE™ His Tag Antibody (GenScript, A00186; 0.2  µg/mL). The same secondary antibody was used for both primary antibodies: polyclonal rabbit anti-mouse immunoglobulins conjugated to horseradish peroxidase (Agilent/DAKO, P026002-2; 1:1000 dilution). Detection was carried out using chemiluminescence. Clarity™ Western ECL substrate (Bio-Rad) was prepared according to the manufacturer’s instructions, and proteins were visualized using a ChemiDoc imaging system (Bio-Rad) after 20 min of substrate incubation.

### Protein glycosylation

The glycosylation of the protein was determined through the periodic acid–Schiff (PAS) staining method. Recombinant Nictaba originating from *E. coli* was added to the gel as a nonglycosylated control [80]. The positive control was asialofetuin from fetal calf serum (Merck, Darmstadt, Germany). For each protein, approximately 30 µg of protein was loaded onto the gel. SDS-PAGE was performed, and the gel was submerged in fixation solution (40% (v/v) methanol and 7% (v/v) acetic acid) for 30 min. Then the fixation solution was replaced with fresh solution and again incubated for 30 min. The latter procedure was performed for a total of four times. The last wash was incubated overnight. The gel was washed twice with fresh fixation solution for 30 min per wash. Subsequently, the gel was submerged in the oxidation solution (1% (v/v) periodic acid and 3% (v/v) acetic acid) for 60 min. The gel was then washed 10 times for 10 min with distilled water. Finally, the gel was transferred to Schiff’s reagent (Merck, Darmstadt, Germany) and incubated in the dark for 60 min, after which the gel was incubated at least five times for 10 min in the reducing solution (0.58% (w/v) sodium metabisulfite and 3% (v/v) acetic acid).

### Affinity gel electrophoresis

The affinity of the full protein was evaluated for a range of polysaccharides through affinity gel electrophoresis [81,82]. Polyacrylamide gels (6% (w/v)) were made containing either 0.3% (w/v) or 0.1% (w/v) polysaccharides (DGA, CMC, galactomannan, β-1,4-galactan) or no substrates. GFP-6xHis was used as a nonbinding negative control. The electrophoresis was performed at 4°C at 120 V. The gels were subsequently stained with Coomassie brilliant blue G-250. The data analysis of the gels was processed through FIJI. The peaks corresponding to the proteins were determined and the relative mobility was evaluated based on the dye front. To evaluate whether the observed differences between the substrate-containing lanes and the control PAGE gel (without substrate) were statistically significant, we assumed a normal distribution with a mean of 0 and a standard deviation corresponding to the measured technical variation from the nonbinding negative control. Observed values were compared against this distribution using a two-sample *t*-test.

### Enzyme activity

#### Screening of potential substrates

The substrate specificity of ATIYA1 was assessed using various compounds. The enzyme reactions contained 1.0% (w/v) of polysaccharide, 0.1 M acetate buffer (pH 5.0), and 0.68 µg/mL purified enzyme. The reactions were incubated at 30°C. The adapted Somogyi-Nelson microplate assay [83] was employed to quantify the release of reducing sugars. In short, a sample was taken and mixed with the copper-carbonate-tartrate reagent in a 1:1 composition. The reaction-copper-carbonate-tartrate mix was incubated at 95°C for 20 min. Subsequently, the incubated mix was combined with half the volume of arsenomolybdate color reagent. The solution was incubated at room temperature for 10 min. The absorbance was measured at 595 nm using a plate reader (Infinite 200, Tecan, Mannedorf, Switzerland).

#### Optimal pH and temperature

The effect of pH on the enzymatic activity of ATIYA1 was determined. The reactions were performed in 0.1 M sodium acetate buffer (pH 3.5–5.6) and 0.1 M potassium phosphate buffer (buffering range 5.8–8.0) at 30°C. The influence of temperature on enzymatic activity was evaluated in 0.1 M sodium acetate buffer pH 5. Each reaction solution contained an appropriate buffer, 0.58 µg purified enzyme /mL reaction and 10 mg/mL DGA.

#### Catalytic efficiency of methylated galacto-oligosaccharides

The catalytic efficiency of the GH5_11 enzyme was checked for different galacto-oligosaccharides. The enzyme reactions consisted of 100 µM of galacto-oligosaccharide, 10 nM enzyme solution, and 0.1 M sodium acetate buffer (pH 5.0). The reactions were incubated at 30°C and samples were taken at distinct time points (0, 5, 10, 20, 60, and 120 min). Three replicates were performed for each enzyme reaction. The reactions were stopped by incubating the samples at 95°C for 5 min. The same volume of ultrapure water was added to the samples and carbohydrates were quantified by high-performance anion-exchange chromatography with a pulsed amperometric detection (HPAEC-PAD) system. A Dionex ICS-6000 (Thermo Fisher Scientific) with a CarboPac PA20 pH stable column was used. Samples were analyzed at a constant flow rate of 0.5 mL/min. For the first 7 minutes an isocratic elution with 25 mM NaOH was used, after which the concentration was linearly increased to 100 mM NaOH at minute 8. While keeping the NaOH concentration constant, the sodium acetate concentration gradually increased from 0 to 50 mM over 5 min and was held constant for 3 min. The sodium acetate concentration was further increased, reaching 200 mM at minute 18. Subsequently, the concentration increased to 500 mM over 30 s and was constant for 3 min, after which the initial buffer composition was restored for 30 s. The column was equilibrated under these conditions for 4.5 min before concluding the run.

The catalytic efficiency (kcat/Km) was determined for the galacto-oligosaccharides using the formula:

$k*t=ln(\frac{\left[S_{0}\right]}{\left[S_{t}\right]})$ with $k=\frac{k_{cat}}{K_{M}}*\left[E\right]$ . (1)

[S_0_], (S_t_) and t representing the initial substrate concentration, the substrate concentration at time t and the time, respectively [38,82,84]. The formula is only valid when $\left[E\right]\ll \left[S\right]\ll K_{M}$ . The validity of the first-order approximation was empirically assessed, confirming that the reaction followed a pseudo-first-order kinetics within the experimental conditions. Subsequently, formula 1 was used to determine the catalytic efficiencies of the enzyme for different galacto-oligosaccharides.

#### Arabinogalactan enzymatic hydrolysis and analysis by polyacrylamide carbohydrate gel electrophoresis (PACE)

Larch arabinogalactan (1 mg) was sequentially digested by different arabinogalactan active enzymes similar to the experiments performed in [16,17]. α-l-arabinofuranosidase (EC 3.2.1.55), exo-β-(1,3)-galactanase (EC 3.2.1.145) and endo-β-(1,6)-galactanase (EC 3.2.1.164) hydrolyses were performed in 50 mM ammonium acetate (pH 6.0) at 37°C for 24 h. The enzymes were deactivated for 5 min at 100°C and then dried in a rotary evaporator. For GH5_11 hydrolysis, samples were resuspended in 100 mM acetate buffer (pH 5.0) to a concentration of 5 mg/mL substrate. The end concentration of protein in the enzyme reaction was 6 µg/mL. The reactions were incubated at pH 5 and 30°C for approximately 10 h. The derivatization of carbohydrates was performed according to an updated protocol [85]. Carbohydrate electrophoresis and PACE gel scanning and quantification were performed as described previously [17]. Control experiments without substrates or enzymes were performed under the same conditions to identify any nonspecific compounds in the enzymes, polysaccharides/cell walls, or labeling reagents.

### Molecular docking and dynamics

The three-dimensional structure of the target protein was obtained from the AlphaFold Protein Structure Database. The model was truncated to retain only the GH5_11 catalytic domain. β-1,6-galacto-oligosaccharide ligands with degrees of polymerization 2, 4, and 6 were generated using the YASARA (version 21.12.19) model builder [86].

To prepare the protein structure for molecular docking, an initial 200 ns molecular dynamics (MD) simulation was performed to equilibrate the glycosylated protein. Protonation states were assigned at pH 5.0 using the APBS biomolecular solvation software suite [87]. Full details of the MD protocol are described below. The glycosylated model was generated by CHARMM. The final frame of the simulation was selected as input for docking.

Molecular docking was conducted using the AutoDock Vina module implemented in YASARA, with default parameters and 50 independent runs per ligand. Docking was performed in the equilibrated protein. The simulation boxes were cuboid and centered around the active site residues. The dimensions were: galactobiose, 20.01 × 20.01 default 20.01 Å; galactotetraose, 23.00 × 23.00 default 23.00 Å; and galactohexaose, 23.00 × 25.00 default 27.00 Å. The most accurate binding poses were selected based on the orientation of the glycosidic bond relative to the catalytic residues (~4 Å).

MD simulations of the unbound protein, its complexes with galactobiose, galactotetraose, and galactohexaose, and of the free β-1,6-galactohexaose were performed using pmemd.cuda in AMBER22 [88]. System setup was performed through the CHARMM-GUI webserver [67,89,90]. The AMBER FF19SB force field [91] was used for the protein, and GLYCAM_06 j [92] for the oligosaccharides. All systems were solvated in a cubic TIP3P water box with a 10 Å buffer and neutralized with Na^+^ and Cl⁻ counterions.

Energy minimization was performed with a maximum of 10,000 steps of steepest descent, using a convergence threshold of 10 kJ/mol. Equilibration was conducted under the NVT and NPT ensembles, both at 300 K using a Langevin thermostat to control temperature. Pressure in the NPT ensemble was regulated using an isotropic Monte Carlo barostat [93]. Production simulations were carried out under NPT conditions for 200 ns (unbound model) and 250 ns (ligand-bound systems) with a 2 fs timestep. SHAKE constraints were applied to all covalent bonds involving hydrogen atoms [94]. Long-range electrostatic interactions were calculated using the Particle Mesh Ewald method with a 9 Å cutoff [95].

Trajectory analyses were performed using CPPTRAJ and VMD [96,97], including root-mean-square deviation (RMSD), radius of gyration, intermolecular hydrogen bonding, contact frequency, active-site distances relevant to catalysis, and puckering state assessments. For the contact frequency, residues within 5 Å of the ligand [98] for at least 60% of the simulation time were considered significant [99]. The puckering conformational analysis used a non-interacting galactose ring at the −2 subsite as a negative control. The reference galactose ring was part of the ATIYA1 + β-1,6-galactohexaose system. It does not interact with the catalytic residues or participate in hydrolysis and thus serves as a reference to evaluating conformational preferences imposed by the active site.

Post-processing and data visualization were performed in Python. Statistical analysis of the gyration radius was performed in Python. Assumptions for parametric tests were evaluated using the Shapiro–Wilk test for normality and the Levene’s test for homogeneity of variances. When the assumptions were not met, nonparametric analyses were conducted employing Kruskal–Wallis test followed by pairwise Mann–Whitney U tests. Bonferroni correction was applied for multiple hypothesis testing correction.

## Supplementary material

online supplementary material 1.

online supplementary table 1.

online supplementary table 2.

## Data Availability

All data are available from the authors upon request. All structures used for the structural analysis and MD simulations, including the ATIYA1 and reference proteins, as well as the equilibrated ATIYA1 structure and associated protein complexes, were stored in ModelArchive [100]. The models are available in ModelArchive (www.modelarchive.org) with the accession code ma-atiya1-gk (link: http://www.modelarchive.org/doi/10.5452/ma-atiya1-gk).

## Open Access

This article has been published open access under our Subscribe to Open programme, made possible through the support of our subscribing institutions, learn more here: https://portlandpress.com/pages/open_access_options_and_prices#conditional

## Abbreviations

AG: arabinogalactan
AGP: arabinogalactan protein
BSA: bovine serum albumin
CAZYme: carbohydrate-active enzyme
CMC: carboxymethylcellulose
DGA: mildly acid digested gum arabic
DP: degree of polymerization
GFP: green fluorescent protein
GH5: glycosyl hydrolase family 5
GH: glycosyl hydrolase
GH5_11: glycosyl hydrolase family 5 subfamily 11
HPAEC-PAD: high performance anion-exchange chromatography with a pulsed amperometric detection
IMAC: immobilized metal affinity chromatography
MD: molecular dynamics
PACE: polyacrylamide carbohydrate gel electrophoresis
PAS: periodic acid-Schiff
PCR: polymerase chain reaction
RFP: red fluorescent protein
RMSD: root-mean-square deviation
SDS-PAGE: sodium dodecyl sulfate-polyacrylamide gel electrophoresis
SP: signal peptide
TIM: triose-phosphate isomerase
WB: Western blot
kDa: kilodalton
pI: isoelectric point
