# Supplementary material for: Expression and characterization of an endo-β-1,6-galactanase from Arabidopsis thaliana
Source: Biochem J. 2025 Dec 17;482(24):1935–57. doi: 10.1042/BCJ20253301 (PMC12751061; doi:10.1042/BCJ20253301)
Supplement: online supplementary material 1. [file bcj-482-24-BCJ20253301-s001.pdf]

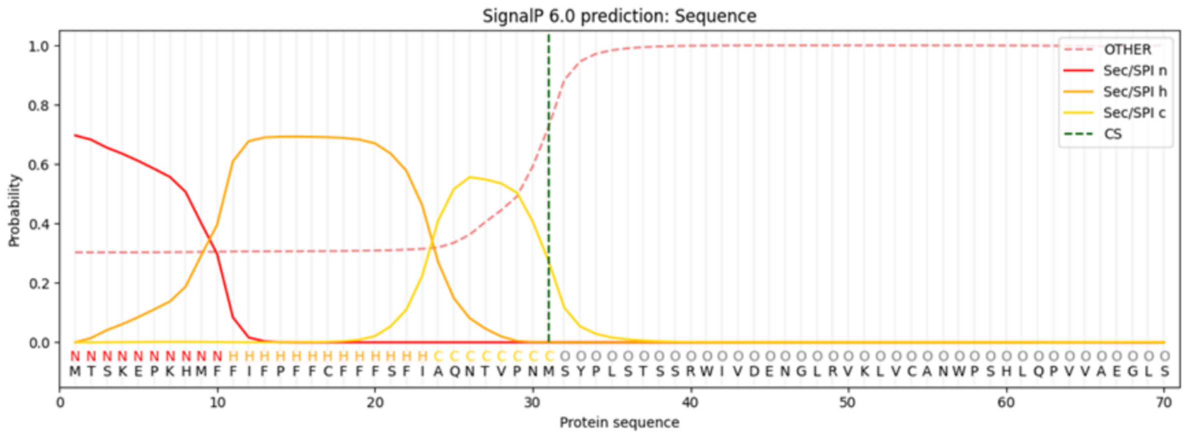

Supplementary Figure 1: Output for the prediction of the signal peptide by SignalP v6.0 . 'Other' annotates the absence of a signal peptide; 'Sec/SPI n', 'Sec/SPI h' and 'Sec/SPI c' represent respectively the N-terminal, the center hydrophobic and the C-terminal region of the signal peptide. 'CS' denotes the last amino acid residue part of the signal peptide.

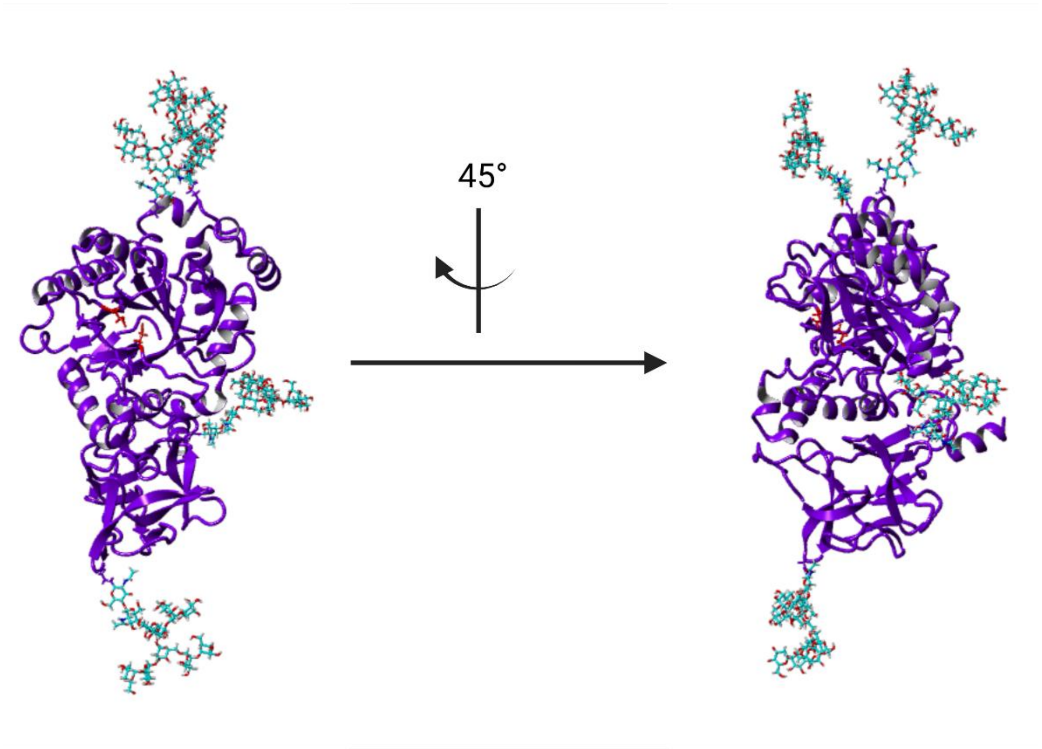

Supplementary Figure 2: Overview of positions for 4 putative N-glycosylation sites predicted for ATIYA1 through the Plant PTM viewer. The protein backbone is coloured purple with the catalytic residues being red. The glycosylation is represented in cyan. The N-glycans depicted of the high-mannose type.

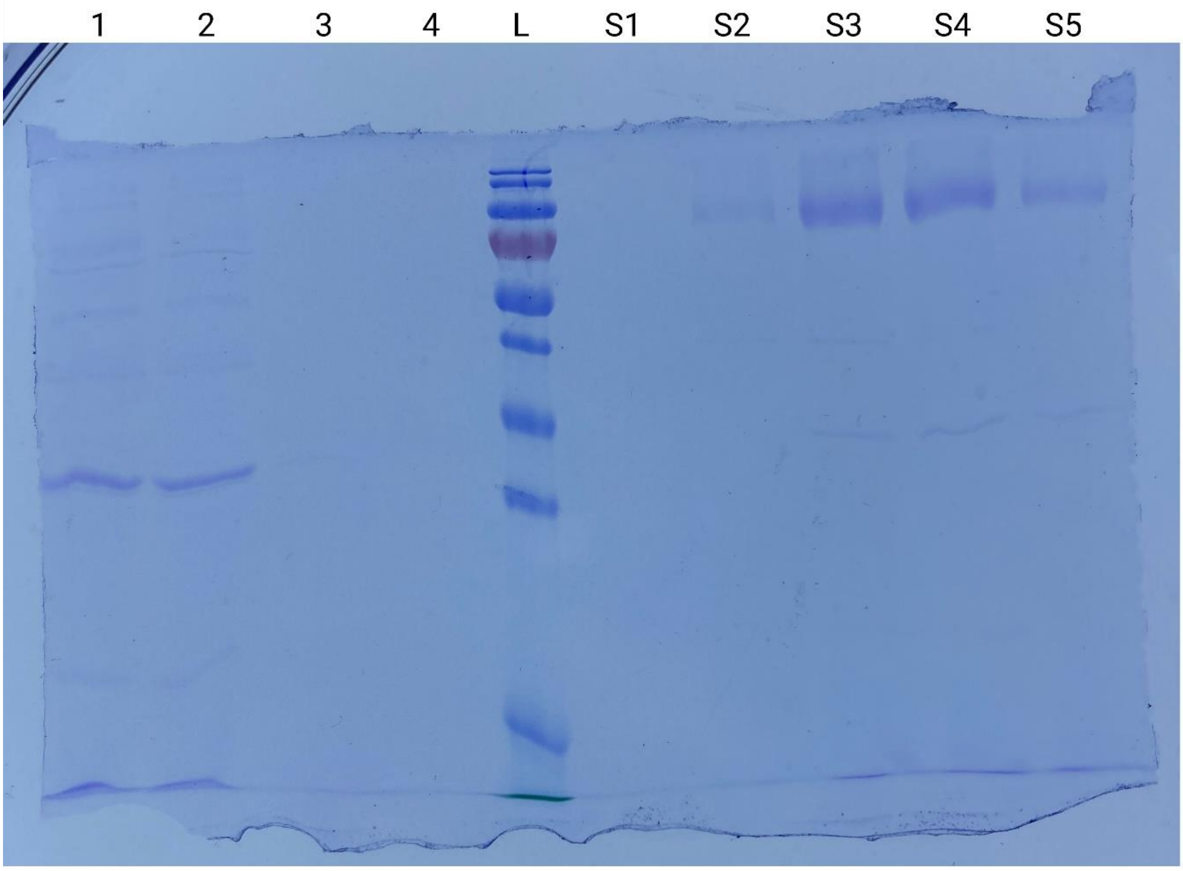

Supplementary Figure 3.1: SDS-PAGE gel (15% polyacrylamide gel) after Coomassie staining showing the different fractions collected during immobilized metal affinity chromatography (IMAC). Lane 1: loading sample; Lane 2: flow-through; Lane 3: wash fraction 1; Lane 4: wash fraction 2; Lanes S1–S4: elution fractions. Lane L: molecular weight marker. 15 µL of each protein sample was loaded into each lane. ***Lane S3*** *and the ladder were used to generate* ***Figure 4 panel A CBS 1****.*

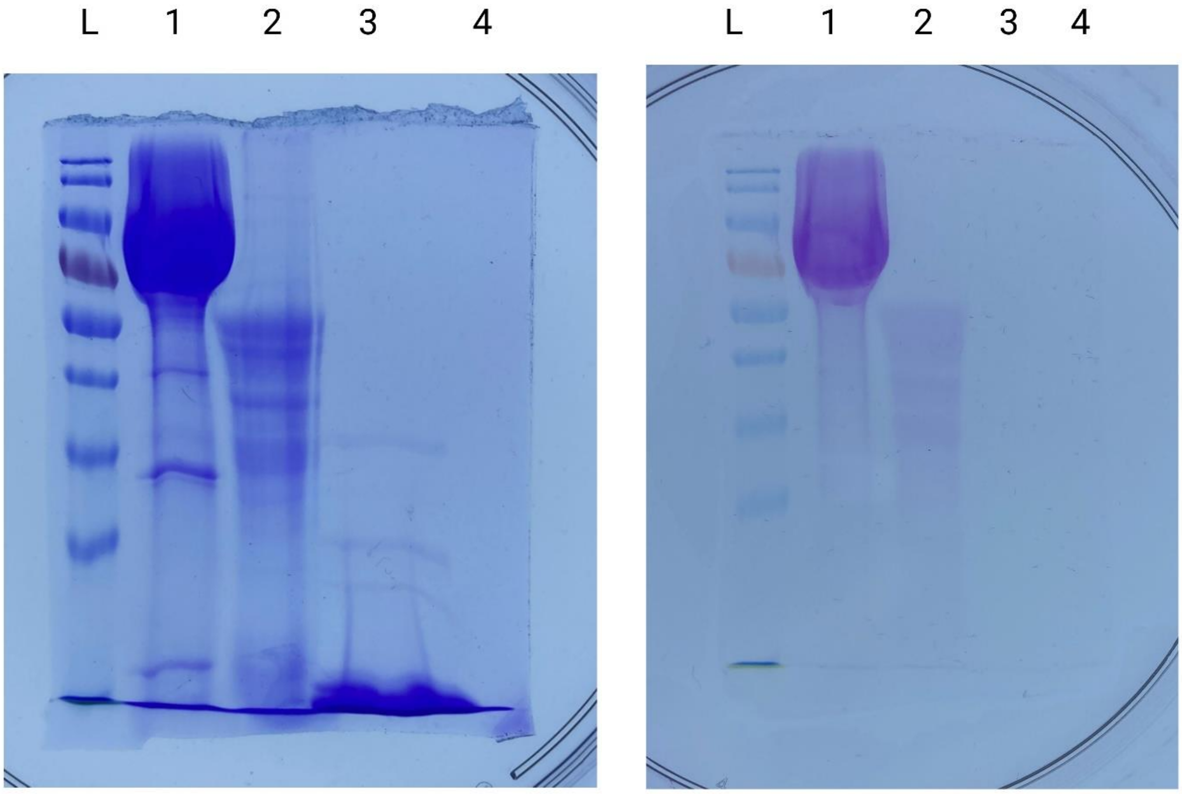

Supplementary Figure 3.2: SDS-PAGE (15% polyacrylamide gel) followed by Coomassie staining (left) and Periodic acid–Schiff (PAS) staining (right). Lane 1: ATIYA1; Lane 2: asialofetuin from fetal calf serum (positive control); Lane 3: recombinant Nictaba expressed in E. coli (negative control); Lane 4: empty. Lane L: molecular weight marker. A total of 30 µg of protein was loaded per lane. The same samples were loaded for both analyses. Samples were loaded on a single SDS-PAGE gel, which was cut in half after electrophoresis and processed separately for Coomassie blue staining and PAS detection. ***Lane 1*** *and the ladder from both halves were used to generate* ***Figure 4, Panel A*** *(lanes* ***CBS 2*** *and* ***PAS 3****).*

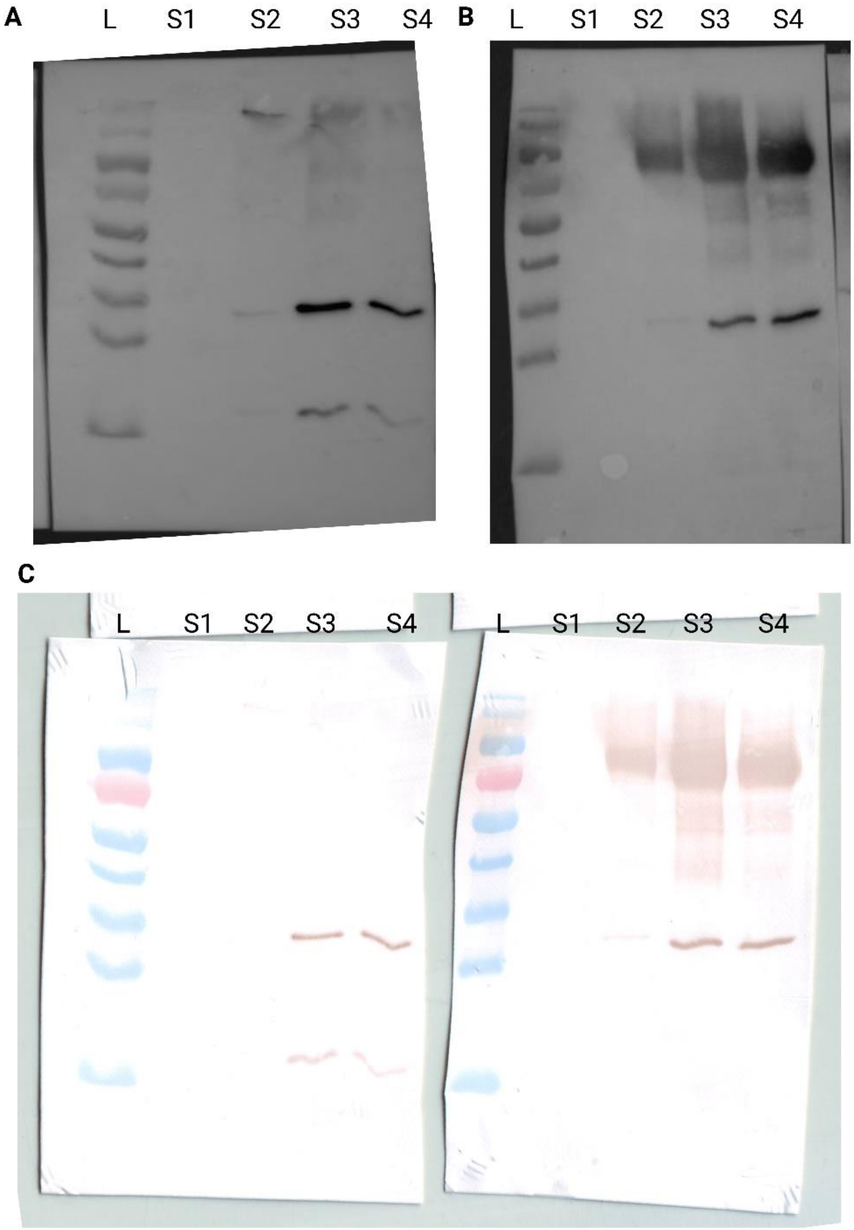

Supplementary Figure 3.3: Western blot analysis using chemiluminescence (Panel A and Panel B) and DAB staining (Panel C) to detect the mCherry tag (Panel A and left blot in Panel C) and the His6-tag (Panel B, right blot in Panel C), showing fractions collected during immobilized metal affinity chromatography (IMAC). Lanes S1–S3: elution fractions; Lane L: molecular weight marker. The blots shown in Panels A and B are identical to those in Panel C. After chemiluminescence the blots were rinsed with water and subjected to DAB staining. Samples were loaded on a single SDS-PAGE gel, transferred to a membrane and the blot was cut in half for separate detection using anti-mCherry and anti-His6 antibodies. ***Lanes S3*** *from* ***Panels A and B*** *and their corresponding ladders were used to generate* ***Figure 4, Panel A*** *(lanes* ***H1*** *and* ***R1****).*

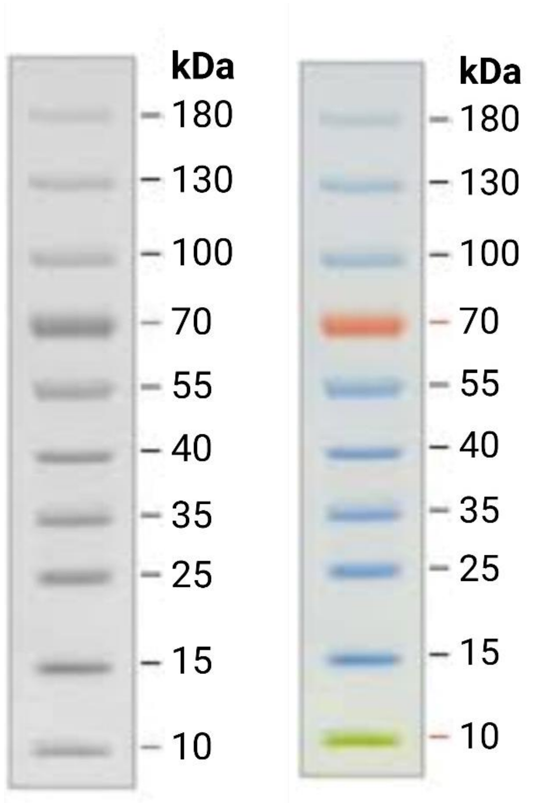

Supplementary Figure 3.4: Prestained protein ladder (PageRuler™ Prestained Protein Ladder, Thermo Fisher Scientific) used as a molecular weight reference for both SDS-PAGE and Western blot analyses. Images on the right and left show the ladder in color and grayscale, respectively. Molecular weights (kDa) are indicated alongside the corresponding bands.

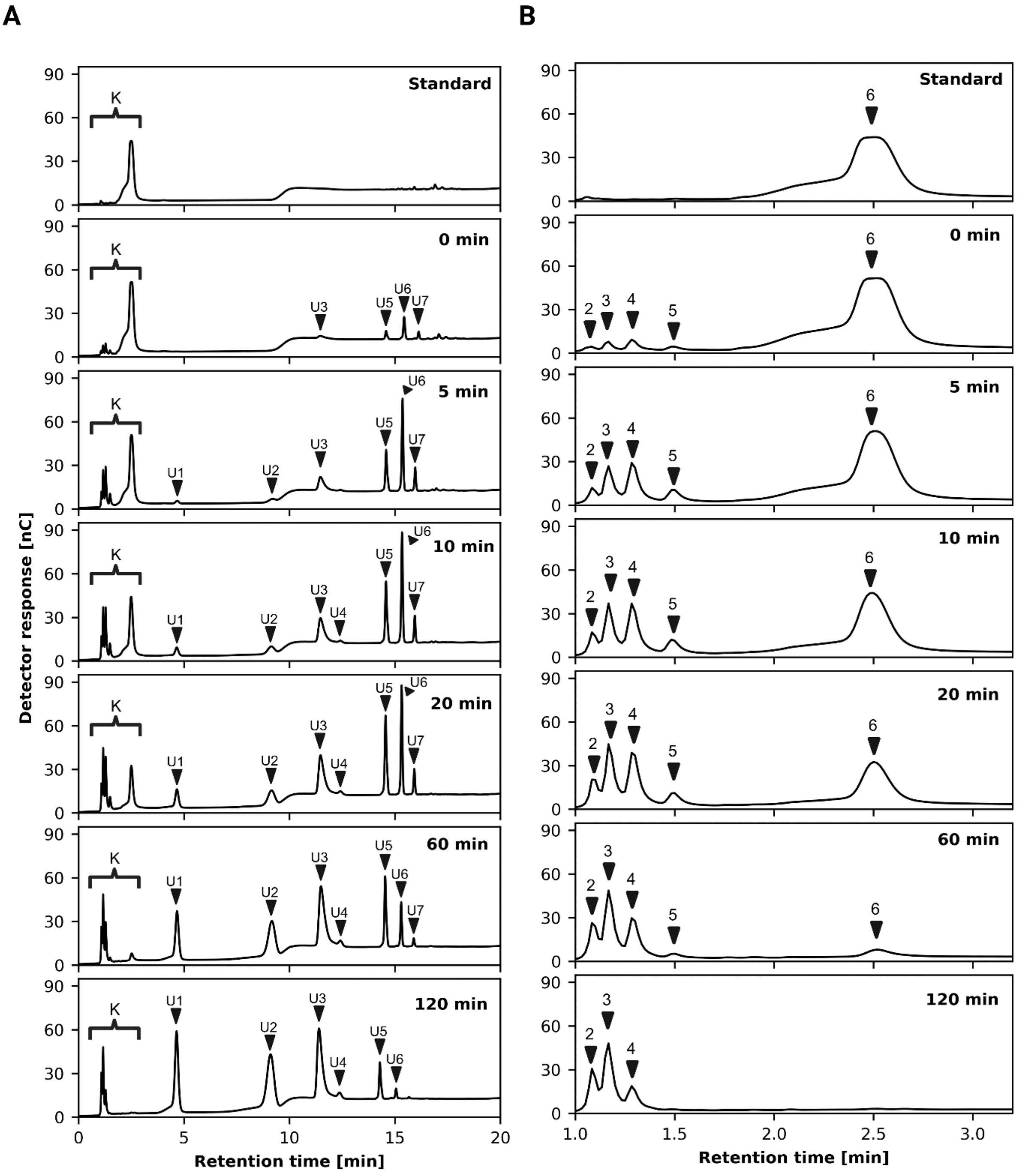

Supplementary Figure 4: HPAEC-PAD chromatograms showing the enzymatic hydrolysis of methylated β-1,6-galactohexaoside by ATIYA1 are presented in (A) and (B) for the substrate alone (Standard) and after 0, 5, 10, 20, 60 and 120 min of incubation. 0 min of incubation corresponds to samples in which enzyme was added and immediately quenched. Panel (A) shows the full chromatograms. Reaction-dependent peaks are labeled as K (known) and U1-7 (unknown). Panel (B) provides a detailed view of the chromatographic region K corresponding to the elution of methylated oligosaccharides (1-3 min). Peak identities were assigned based on retention times of reference standards. Arrows 1-6 indicate distinct degrees of polymerization.

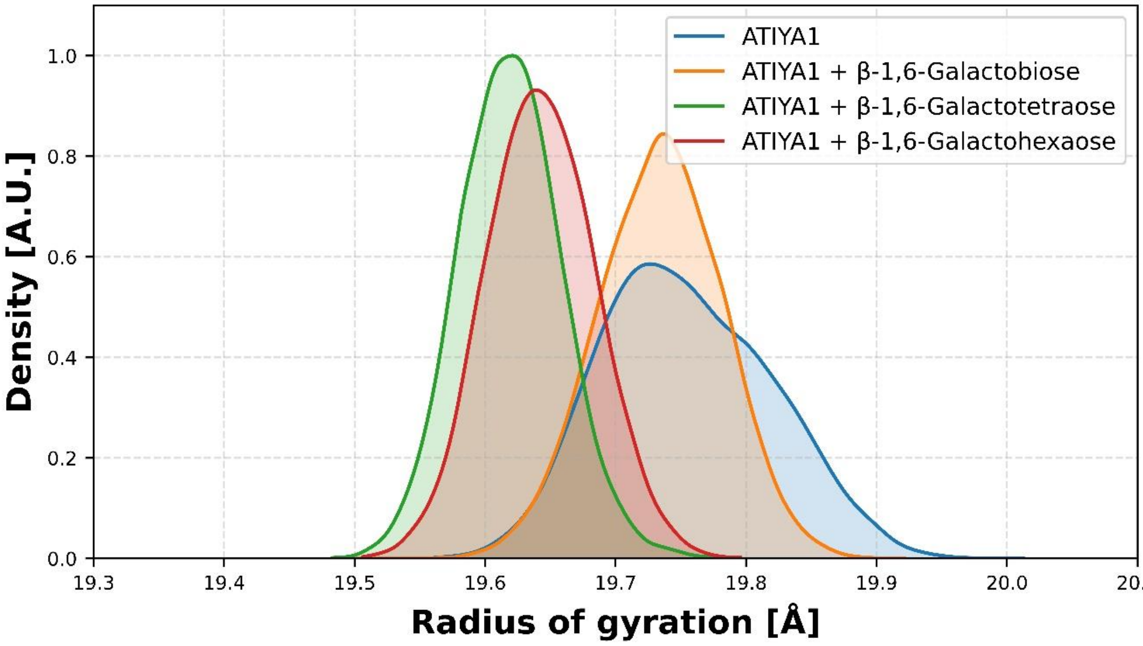

Supplementary Figure 5: Density distribution of the radius of gyration for the distinct free and ligand-bound complexes during the MD simulation. The density is expressed as arbitrary units (A.U.).

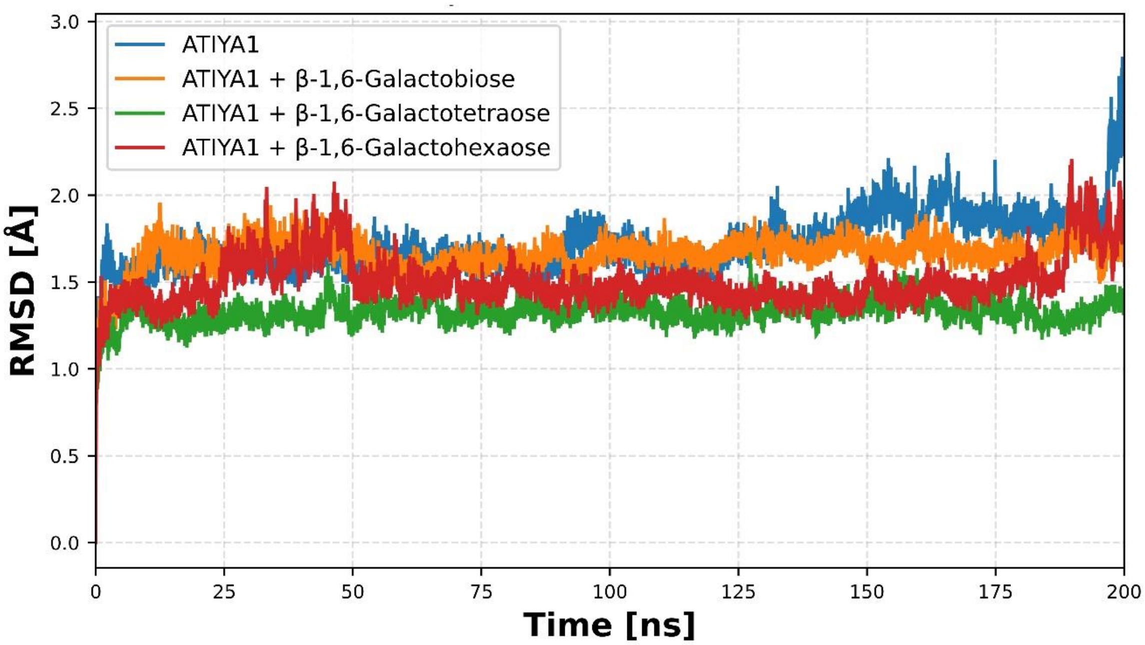

Supplementary Figure 6: RMSD plot of the protein backbone as a function of time for the different protein-ligand complexes.

A

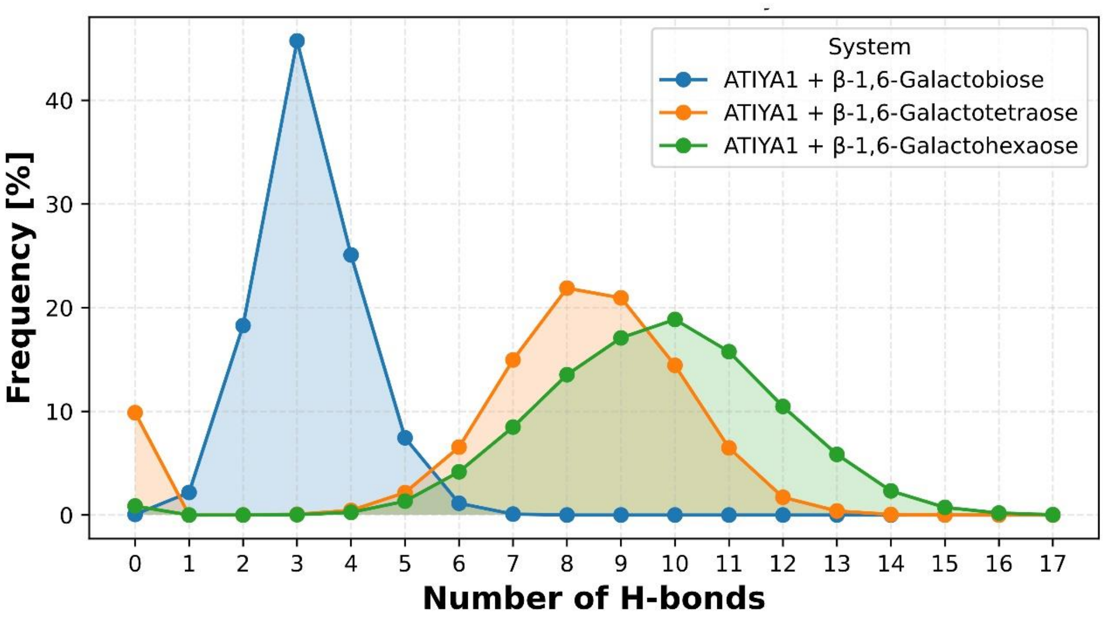

B

Complex	Number of hydrogen bonds
**ATIYA1 + β-1,6-galactobiose**	3.21 ± 0.95
**ATIYA1 + β-1,6-galactotetraose**	7.63 ± 2.94
**ATIYA1 + β-1,6-galactohexaose**	9.65 ± 2.25

Supplementary Figure 7: Frequency distribution of the number of hydrogen bonds detected during the MD simulations (A) along with the average number of hydrogen bonds detected (± the standard deviation) for each complex (B).

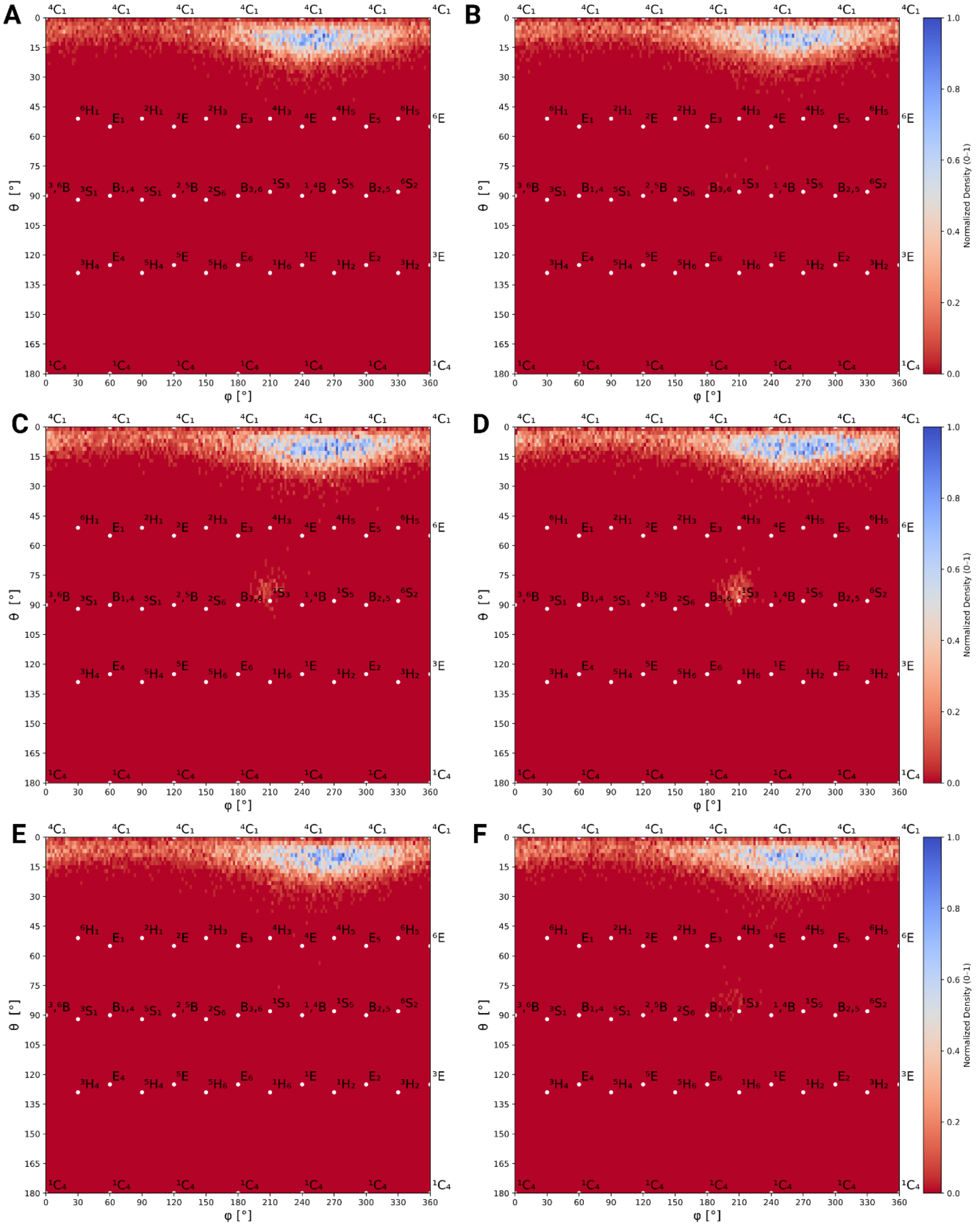

Supplementary Figure 8: Distribution of ring puckering conformations (Cremer–Pople θ–φ maps) for each galactose residue in the MD simulation of free β-1,6-galactohexaose. Panels (A–F) represent residues from the reducing end (A) toward the non-reducing end (F).

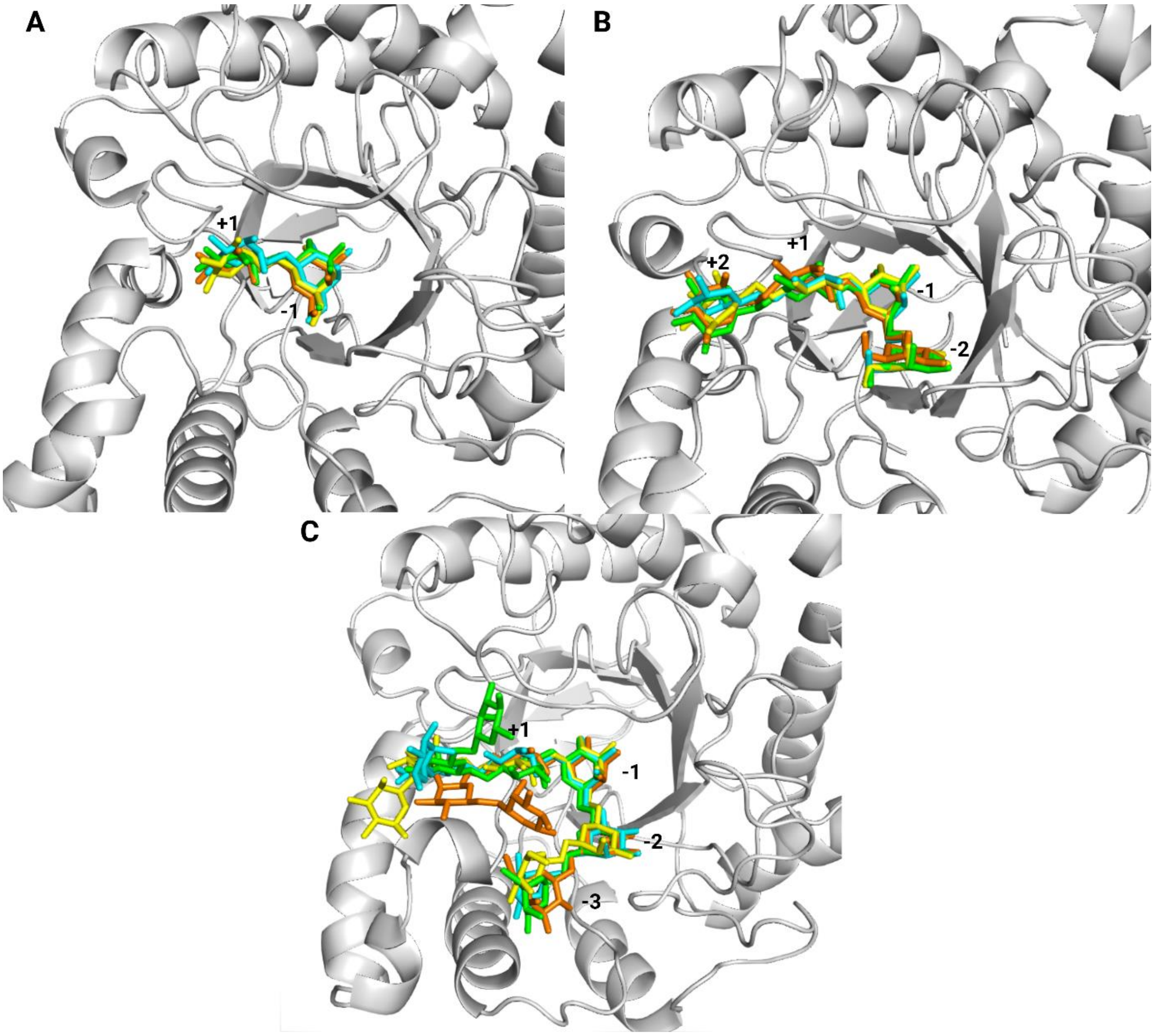

Supplementary Figure 9: Overview of the MD simulations showing the time-evolution of DP2 (A), DP4 (B), and DP6 (C) within the binding groove. The protein backbone (gray) from the 50-ns frame is shown as a reference structure to minimize visual clutter. Ligand conformations extracted at 50 ns (green), 100 ns (cyan), 150 ns (yellow), and 250 ns (orange) are overlaid to illustrate the progression of each trajectory. DP4 maintains a consistent binding mode throughout the simulation, whereas DP6 exhibits greater positional variation, particularly around subsites +2 and +3.

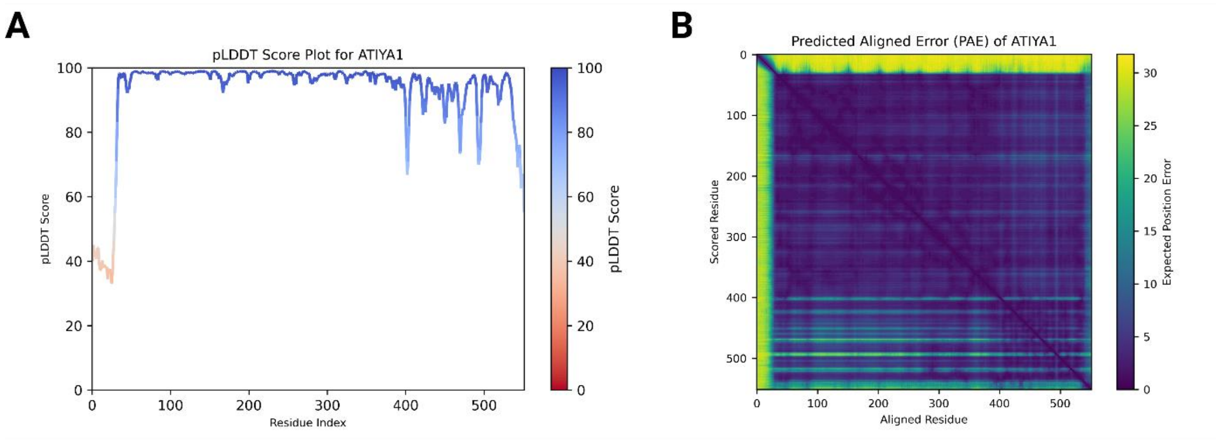

Supplementary Figure 10.1: Model quality measures of the ATIYA1 model obtained from the AlphaFold Protein Structure Database. Panel (A) shows predicted local distance difference test (pLDDT) scores, and panel (B) displays predicted aligned error (PAE) values for the structure.

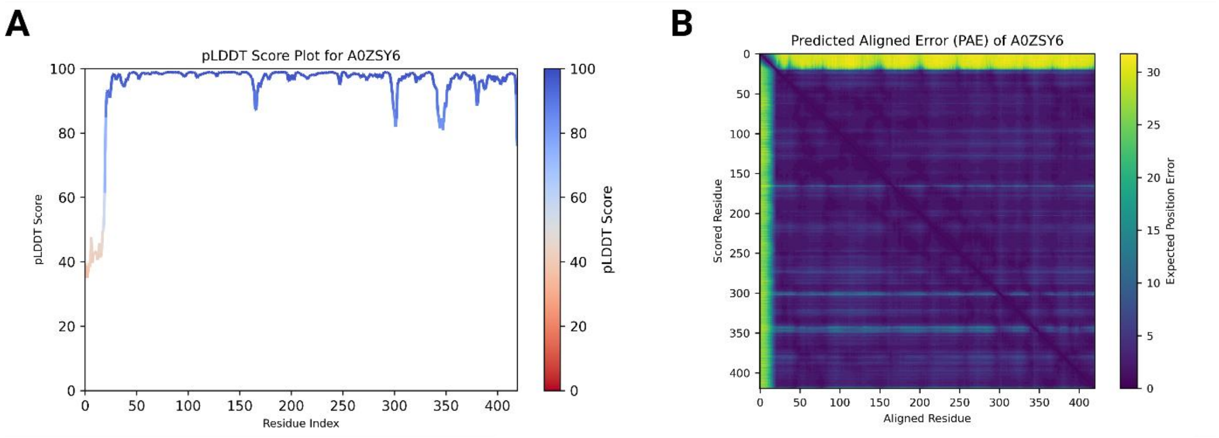

Supplementary Figure 10.2: Model quality measures of the A0ZSY6 model obtained from the AlphaFold Protein Structure Database. Panel (A) shows predicted local distance difference test (pLDDT) scores, and panel (B) displays predicted aligned error (PAE) values for the structure.

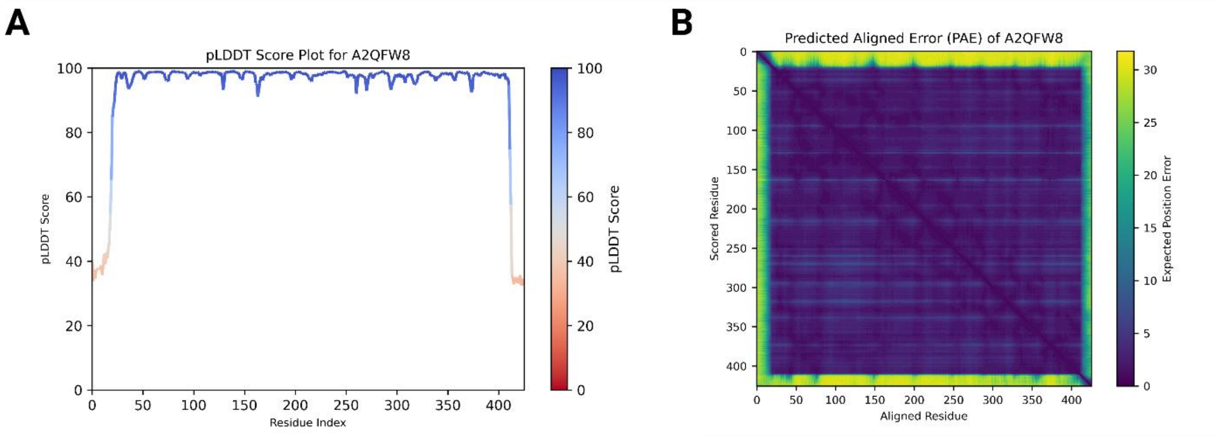

Supplementary Figure 10.3: Model quality measures of the A2QFW8 model obtained from the AlphaFold Protein Structure Database. Panel (A) shows predicted local distance difference test (pLDDT) scores, and panel (B) displays predicted aligned error (PAE) values for the structure.

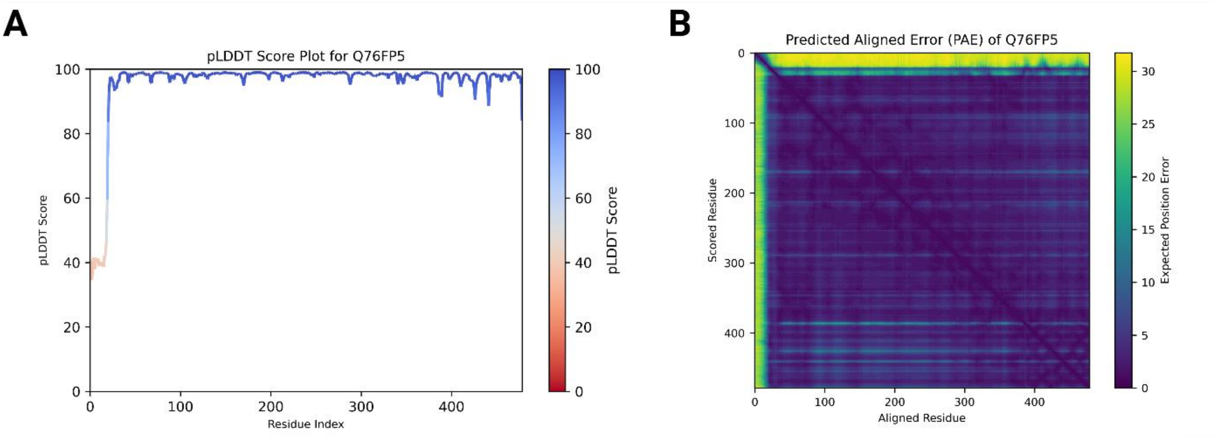

Supplementary Figure 10.4: Model quality measures of the Q76FP5 model obtained from the AlphaFold Protein Structure Database. Panel (A) shows predicted local distance difference test (pLDDT) scores, and panel (B) displays predicted aligned error (PAE) values for the structure.

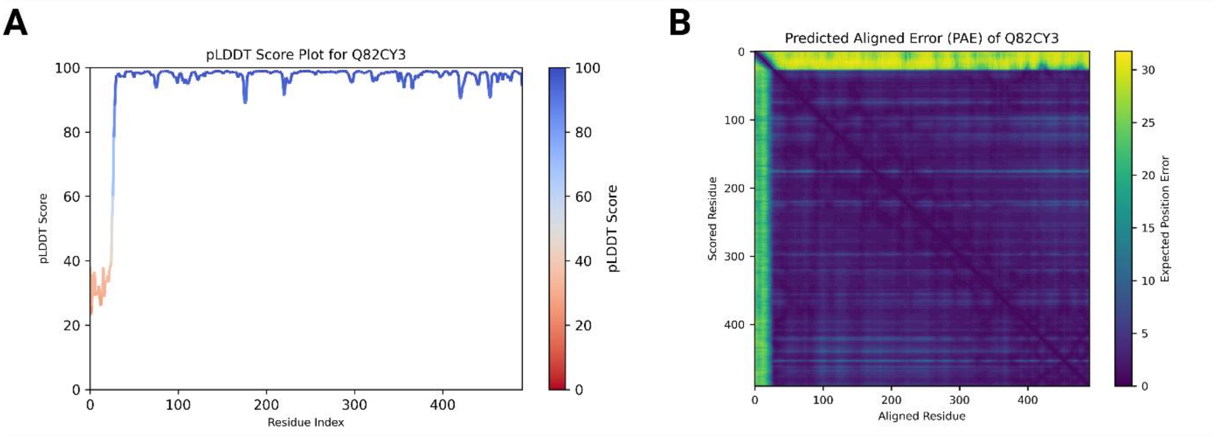

Supplementary Figure 10.5: Model quality measures of the Q82CY3 model obtained from the AlphaFold Protein Structure Database. Panel (A) shows predicted local distance difference test (pLDDT) scores, and panel (B) displays predicted aligned error (PAE) values for the structure.

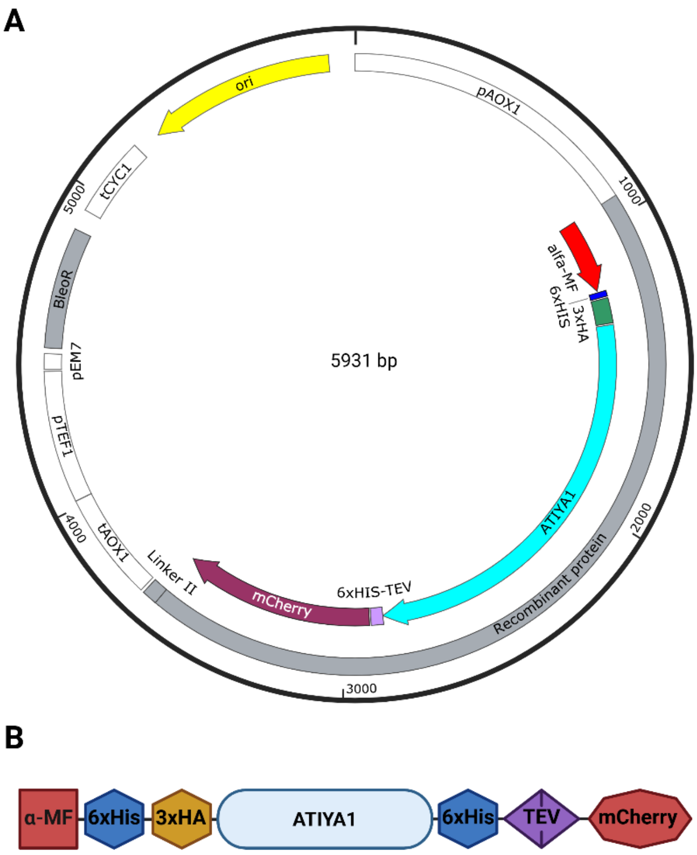

Supplementary Figure 11: Schematic representation of the plasmid for expression and the recombinant protein sequence from start to stop codon. The protein starts with an α-Mating Factor (α-MF/alpha-MF) from Saccharomyces cerevisiae which was present in the backbone of the pPICZA-α vector. This was followed by two purification tags namely His_6-tag (6xHis) and a HA_3-tag. Subsequently, the full mature ATIYA1 sequence was added. Another His_6-tag followed by a TEV-protease cleavage site (TEV) and an mCherry tag was added, allowing to follow the expression of the protein of interest. The figure was made using BioRender.
