# Supplementary material for: Expression and characterization of an endo-β-1,6-galactanase from Arabidopsis thaliana
Source: Biochem J. 2025 Dec 17;482(24):1935–57. doi: 10.1042/BCJ20253301 (PMC12751061; doi:10.1042/BCJ20253301)
Supplement: online supplementary table 1. [file bcj-482-24-BCJ20253301-s002.pdf]

Supplementary Table 1: Interacting residues with the ligand for the different MD simulations. Conserved or equivalent residues based on the ESPRIPT analysis were highlighted in orange.

ATIYA1 + β-1,6-galactobiose		ATIYA1 + β-1,6-galactotetraose		ATIYA1 + β-1,6-galactohexaose	
**Residue**	**Occupancy (%)**	**Residue**	**Occupancy (%)**	**Residue**	**Occupancy (%)**
HIS29	99.95	HIS29	90.1	HIS29	99.13
ARG59	99.86	ARG59	90.11	ARG59	99.01
THR61	61.9	HIS129	90.13	THR61	62.52
HIS129	100	TRP135	90.13	HIS129	99.13
TRP135	99.6	CYS136	90.13	TRP135	99.13
CYS136	100	CYS137	90.13	CYS136	99.13
CYS137	99.59	ALA138	85.92	CYS137	99.13
ASN180	100	ASN139	72.18	ALA138	98.15
GLU181	100	ASN180	90.13	ASN139	77.03
PHE219	100	GLU181	90.13	ASN180	99.13
HIS244	99.97	SER218	73.53	GLU181	99.13
TYR246	100	PHE219	90.13	SER218	70.58
PHE248	99.78	HIS244	90.13	PHE219	99.13
SER249	93.94	TYR246	90.13	HIS244	98.85
GLU288	100	PHE248	90.12	TYR246	99.13
TRP321	100	SER249	90.13	SER247	69.94
TYR327	88.94	ASP250	75.57	PHE248	99.13
GLU338	95.34	GLY251	68.85	SER249	98.98
TYR340	98.98	GLU288	90.13	ASP250	94.68
		TRP321	90.13	GLY251	78.14
		TYR327	90.1	GLU288	99.13
		ARG330	90.12	ASP292	75.35
		GLU338	90.13	ASN297	79.91
		TYR339	88.38	ASP300	74.7
		TYR340	90.13	TRP321	99.13
				TYR327	99.1
				ARG330	99.13
				GLU338	99.13
				TYR339	97.95
				TYR340	99.13
