# Supplementary material for: Expression and characterization of an endo-β-1,6-galactanase from Arabidopsis thaliana
Source: Biochem J. 2025 Dec 17;482(24):1935–57. doi: 10.1042/BCJ20253301 (PMC12751061; doi:10.1042/BCJ20253301)
Supplement: online supplementary table 2. [file bcj-482-24-BCJ20253301-s003.pdf]

Supplementary Table 2: pH and temperature optima from different characterized endo-β-1,6-galactanases (EC 3.2.1.164) from literature.

**Subfamily**	**EC**	**Protein name**	**Origin**	**pH optimum**	**T optimum**	**Substrate**
GH5_16	3.2.1.164	FoGal1	*Fusarium oxysporum*	3.5	50°C	Larch AG
GH30_5	3.2.1.164	Sa1,6Gal5A	*Streptomyces avermitilis*	5.5	37°C	β-1,3/1,6-galactan from *P. zopfii*
GH30_5	3.2.1.164	Nc6GAL	*Neurospora crassa*	3-4	60°C	β-1,3/1,6-galactan from *P. zopfii*
GH30_?	3.2.1.164	PoGal30	*Penicillium oxalicum*	4	40°C	DGA
